# Deuterostome Genomics: Lineage-Specific Protein Expansions That Enabled Chordate Muscle Evolution

**DOI:** 10.1093/molbev/msy002

**Published:** 2018-01-08

**Authors:** Jun Inoue, Noriyuki Satoh

**Affiliations:** Marine Genomics Unit, Okinawa Institute of Science and Technology Graduate University, Onna, Okinawa, Japan

**Keywords:** muscle structural proteins, gene duplication, lineage-specific expansion, chordate evolution, phylogenetics

## Abstract

Fish-like larvae were foundational to the chordate body plan, given the basal placement of free-living lancelets. That body plan probably made it possible for chordate ancestors to swim by beating a tail formed of notochord and bilateral paraxial muscles. In order to investigate the molecular genetic basis of the origin and evolution of paraxial muscle, we deduced the evolutionary histories of 16 contractile protein genes from paraxial muscle, based on genomic data from all five deuterostome lineages, using a newly developed orthology identification pipeline and a species tree. As a result, we found that more than twice as many orthologs of paraxial muscle genes are present in chordates, as in nonchordate deuterostomes (ambulacrarians). Orthologs of paraxial-type *actin* and *troponin C* genes are absent in ambulacrarians and most paraxial muscle protein isoforms diversified via gene duplications that occurred in each chordate lineage. Analyses of genes with known expression sites indicated that some isoforms were reutilized in specific muscles of nonvertebrate chordates via gene duplications. As orthologs of most paraxial muscle genes were present in ambulacrarians, in addition to expression patterns of related genes and functions of the two protein isoforms, regulatory mechanisms of muscle genes should also be considered in future studies of the origin of paraxial muscle.

## Introduction

**Fig. 1.**
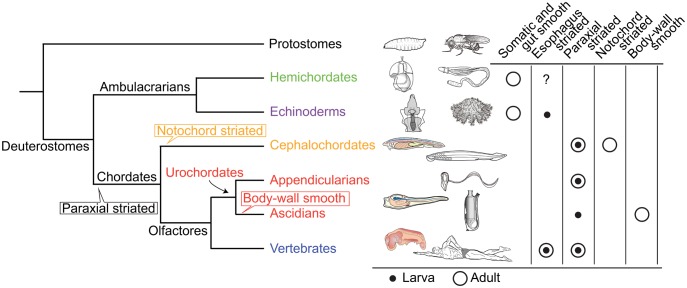
Phylogenetic relationships of major deuterostome lineages (Holland 1996; Simakov et al. 2015) and distribution of somatic and visceral muscle types (Schmidt-Rhaesa 2007). In hemichordates, most musculature exhibits smooth muscle structure (Benito and Pardos 1997) although the possible presence of esophagus striated muscles is suggested (Ceresa Castellani and Saita 1974). In echinoderms, histologically, muscle generally more closely resembles vertebrate smooth muscle than skeletal muscle (Garcia-Arraras and Dolmatov 2010). Striated structure is reported in brachial muscles of crinoids and the base of esophageal muscle of sea urchin larvae (Burke 1981). In cephalochordates, in addition to segmented myotomal musculature composed of striated muscle, notochord is filled with modified striated muscle cells (Holland 1996). In urochordates, appendicularians maintain the tail, consisting of a notochord, a nerve cord, and double bands of muscle cells throughout their lives (Nishino et al. 2000), whereas ascidians lose the larval tail and its muscle cells and form body-wall muscle in the trunk during metamorphosis into sessile adults (Burighel and Cloney 1997). Vertebrates possess skeletal and esophagus (Shiina et al. 2005) striated muscles, and visceral smooth muscles (Marieb and Hoehn 2015). Balloons in the species tree indicate phylogenetic positions of specific muscle origins inferred from the presence or absence of chordate muscle types (for discussion of visceral smooth muscle, see Oota and Saitou 1999; Brunet et al. 2016).

Chordates comprise three phyla (fig. 1), Cephalochordata, Urochordata (Tunicata), and Vertebrata (Satoh et al. 2014). All share characteristic features such as a notochord, a hollow dorsal neural tube, somites, and a postanal tail (Satoh 2016). Chordates originated from deuterostome ancestor(s) shared with the Ambulacraria, which is comprised of Hemichordata and Echinodermata (Satoh 2008; Swalla and Smith 2008). Chordate origins and evolution have been debated and discussed for >150 years (Gee 1996), ever since Charles Darwin’s proposal of the origin of species by means of natural selection (Darwin 1859). Recent studies in molecular phylogeny, evolutionary developmental biology (evo-devo), comparative genomics, and other fields, have advanced our understanding of molecular mechanisms underlying chordate evolution (see recent reviews of Diogo et al. 2015; Green et al. 2015; Holland et al. 2015; Lowe et al. 2015). Several evolutionary scenarios of chordate evolution, including the enteropneust hypothesis and inversion hypothesis, have been proposed and vigorously debated (Holland et al. 2015; Lowe et al. 2015).

We previously proposed a hypothesis of chordate origins, in which we emphasized that development of fish-like or tadpole-like larvae is essential to understand the origin of chordates (Satoh 2003, 2014, 2016). Among deuterostomes, ambulacrarian larvae such as echinoderm pluteus and hemichordate tornaria larvae swim by ciliary movement (fig. 1). In contrast, chordate larvae, such as cephalochordate fish-like and urochordate tadpole-like larvae, swim by beating their tails using bilaterally located muscles (hereafter, paraxial striated muscles). Tail-beating locomotion was presumably more effective to capture prey. Chordate characters, such as a notochord, a hollow, dorsal neural tube, somites, and a postanal tail, all evolved in concert with changes in larval locomotion. In order to better understand chordate origins and evolution, we have to determine how these chordate-specific characters arose. In 2015, genomes of two hemichordates, a direct developer, *Saccoglossus kowalevskii*, and an indirect developer, *Ptychodera flava*, were decoded (Simakov et al. 2015). Genomes have been decoded for a urochordate, *Ciona intestinalis* (Dehal et al. 2002), a cephalochordate, *Branchiostoma floridae* (Putnam et al. 2008), an echinoderm *Strongylocentrotus purpuratus* (Sea Urchin Genome Sequencing Consortium 2006) and various vertebrates, including humans (Venter et al. 2001). We are now able to examine genetic changes associated with chordate evolution in genomes of all five deuterostome phyla.

**Fig. 2.**
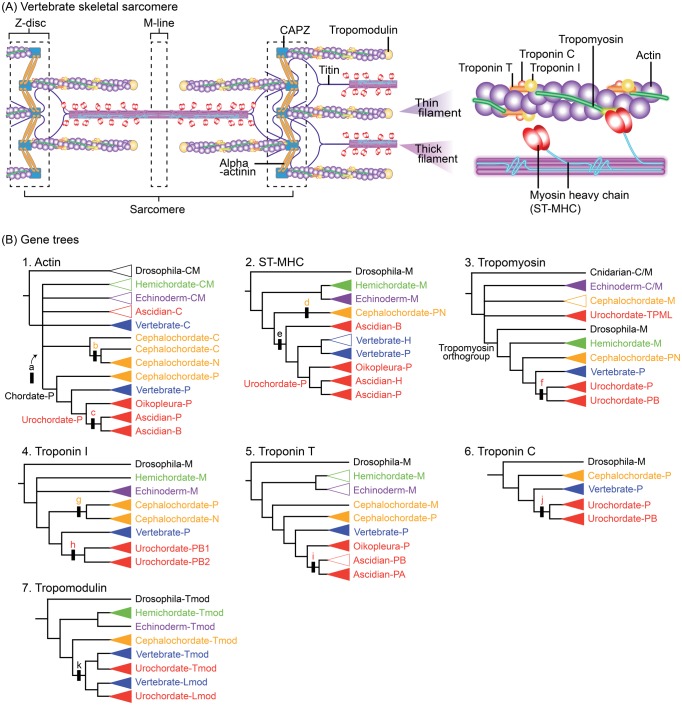
(*A*) Schematic representation of several molecules in vertebrate skeletal sarcomeres. Contraction occurs in response to calcium using troponin–tropomyosin regulatory mechanisms (Schmidt-Rhaesa 2007; Marieb and Hoehn 2015). (*B*) Schematic of estimated muscle protein gene trees (supplementary fig. S2*A*–*G*, Supplementary Material online). Black bars with lower case letters indicate gene duplication events. Upper case letters in clade names indicate inferred expressions of ancestral genes (A, adult; B, body-wall muscle; C, cytoplasm; C/M, cytoplasm or muscle; H, heart; Lmod, leiomodin; M, muscle; N, notochord muscle; P, paraxial muscle; Tmod, tropomodulin; TPML, tropomyosin-like). Triangles indicate gene grouping in which monophyly is supported (closed) or not (open).

Here, we investigated molecular modifications in muscle proteins that might have been involved in changes of larval swimming modes. Vertebrate muscles are classified as striated (skeletal and cardiac) and smooth muscles (Schmidt-Rhaesa 2007). Due to the absence of clearly homologous muscle in ambulacrarians (fig. 1), paraxial muscles (skeletal muscles in vertebrates) are considered a chordate innovation. The contractile apparatus of paraxial muscle, however, centers on actomyosin (fig. 2*A*) which originated in stem eukaryotes (Brunet et al. 2016), and on accessory proteins of premetazoan origin (Steinmetz et al. 2012). Therefore, we focus on major constituents of paraxial muscle proteins with identified human or Drosophila orthologs (Steinmetz et al. 2012). Using accurate tree-based orthology estimation (supplementary fig. S1, Supplementary Material online), we inferred the evolutionary pattern of gene duplication by analyzing whether molecular changes occurred at the base of chordate evolution or independently in each lineage of the three chordate taxa.

## Results

### Semiautomated Pipeline Analysis for Deuterostome Genome Data

To explore the molecular basis of diversification of chordate paraxial muscle, we identified orthologous and paralogous relationships among deuterostome genes (involving gene duplications) by conducting rigorous phylogenetic and rearrangement analysis using a species tree for muscle gene sequences retrieved from genomes of 14 deuterostomes (supplementary table S1, Supplementary Material online). Homologous gene clades identified by our analytical pipeline were regarded as “orthogroups,” including established orthologs of humans and *Drosophila* (or *C. elegans*) (Steinmetz et al. 2012). Using human and nonvertebrate protein-coding gene sequences as representatives of deuterostome lineages, we used the following two-step approach in our semiautomated pipeline: ortholog identification by BLAST search and neighbor-joining (NJ) analysis using a 70% bootstrap criterion (BS 70% criterion) (supplementary fig. S1*A*, Supplementary Material online) and duplication estimate by maximum-likelihood (ML) analysis using identified orthologs (supplementary fig. S1*B*, Supplementary Material online). Nonvertebrate query sequences were identified by preliminary analysis using human queries. When gene sequences with known expression sites were available (supplementary fig. S1*B*5, Supplementary Material online), ancestral isoforms and gene duplications producing characteristic isoforms were identified. We focused on genes expressed in paraxial muscle, and in cephalochordate notochord and ascidian body-wall muscle, but not those in cardiac striated and visceral smooth muscle, due to limited expression data in nonvertebrates.

### Gene Trees

These semiautomated analyses were applied to 36 muscle genes (supplementary table S2, Supplementary Material online), and the analysis succeeded in reconstructing gene trees for 16 structural genes that fulfilled our BS70% criterion. To estimate origins of specialized isoforms, seven isoform families were analyzed using additional gene sequences with known expression sites (fig. 2*B*).

#### Actin

Actin, the principal component of thin myofilaments (fig. 2*A*), is one of the most highly conserved proteins among eukaryotes. Chordate paraxial actins are distinguishable from cytoplasmic (Chiba et al. 2003) or notochord actins (Suzuki and Satoh 2000) by comparing diagnostic positions in the amino acid sequences.

By assigning orthologs and their expression profiles (fig. 2*B*1), we found the clade comprising paraxial-type actin genes belonging to cephalochordates, urochordates, and vertebrates (Chordate-P). Although the phylogenetic position of the Chordate-P clade was unstable, the resultant tree indicated that chordate paraxial-type actin was derived from a gene duplication (shown by a in fig. 2*B*1) that occurred in the lineage leading to chordates after the separation of protostomes and deuterostomes. The Cephalochordate-N clade of notochord-type (Cephalochordate-N) actin was nested within a clade consisting of cytoplasmic-type sequences (Cephalochordate-C) with bootstrap support of 61% and 70% (supplementary fig. S2*A*1, Supplementary Material online). Inclusion of cephalochordate notochord isoforms in a clade containing cytoplasmic isoforms indicates that notochord actin was derived from cytoplasmic actin via gene duplication (b in fig. 2*B*1) within the cephalochordate lineage and has an origin independent of paraxial actin (Chordate-P).

Within the Chordate-P clade (fig. 2*B*1), the Cephalochordate-P clade was placed at the basal position and Vertebrate-P and Urochordate-P clades formed a monophyletic group. The Urochordate-P clade split into three clades: Oikopleura-P, Ascidian-P, and Ascidian-B. Considering the bifurcation of the paraxial (Ascidian-P clade) and body-wall type (Ascidian-B) genes after diverging from Oikopleura paraxial type genes (Oikopleura-P), ascidian body-wall type actins were derived from paraxial-type actins via gene duplication (c in fig. 2*B*1) at the base of ascidians. These results indicate a further diversification of actins in the urochordate lineage.

#### Myosin heavy chain, skeletal

As the principal component of thick myofilaments (fig. 2*A*), all vertebrate myocytes express distinct isoforms of the myosin heavy chain: the striated myosin heavy chain ST-MHC (cardiac or skeletal isoforms) and the smooth/nonmuscle myosin heavy chain SM-MHC (Marieb and Hoehn 2015). Previous studies (Steinmetz et al. 2012) suggested that an ancient gene duplication gave rise to the two distinct phylogenetic groups of *MHC* orthologs before the separation of protostomes and deuterostomes.

Our pipeline analysis identified clades of *ST-MHC* genes corresponding to five deuterostome lineages (fig. 2*B*2). Cephalochordate *ST-MHC* genes expressed in the notochord were nested within the Cephalochordate-PN clade, including paraxial muscle genes (supplementary fig. S2*B*3, Supplementary Material online). This indicates that notochord ST-MHC was derived from paraxial muscle via gene duplication (d in fig. 2*B*2) within the cephalochordate lineage. In the Olfactores lineage, *ST-MHC* genes expressed in cardiac muscles (Vertebrate-H and Ascidian H) were consistently placed at the base of vertebrate or ascidian paraxial-type *ST-MHC* genes. In addition, the Ascidian-B clade, including the *C. intestinalis* gene expressed in body-wall muscle, was placed as the sister group of a clade comprising the Vertebrate-P/-H and Urochordate-P clades. The basal placement of the Ascidian-B clade within the Olfactores clades indicates that ascidian body wall-type ST-MHC was derived from paraxial muscle via a gene duplication (e in fig. 2*B*2) that occurred in the stem Olfactores line, as shown in Chiba et al. (2003).

#### Tropomyosin

Tropomyosin is a long-stranded protein that loops around actin chains in thin filaments (fig. 2*A*). By covering the myosin-binding sites of actin molecules, tropomyosin prevents muscle contraction. Biochemical analyses suggest the existence of tropomyosin in muscles of a sea lily (Echinodermata) (Obinata et al. 2014) and an acorn worm (Hemichordata) (Sonobe et al. 2016).

Our pipeline analysis identified a tropomyosin orthogroup (fig. 2*B*3), including mutually monophyletic groups of deuterostome genes, except for those of echinoderms. Although echinoderm genes (the Echinoderm-C/M clade in supplementary fig. S2*C*1, Supplementary Material online) were placed outside the tropomyosin orthogroup, in situ hybridization analysis (Andrikou et al. 2013) and conservation of troponin-T binding sites (supplementary fig. S2*C*4, Supplementary Material online) imply their expression and function in muscles. Within the tropomyosin orthogroup (fig. 2*B*3), the Hemichordate-M clade was placed in the basal position and urochordate genes were divided into two clades, Urochordate-P and -PB. Inclusion of body wall-type muscle genes of *Ciona* in the Urochordate-PB clade (supplementary fig. S2*C*3, Supplementary Material online) suggests that body wall-type tropomyosin was derived from paraxial-type via gene duplication (f in fig. 2*B*3) in the stem of urochordates.

**Fig. 3.**
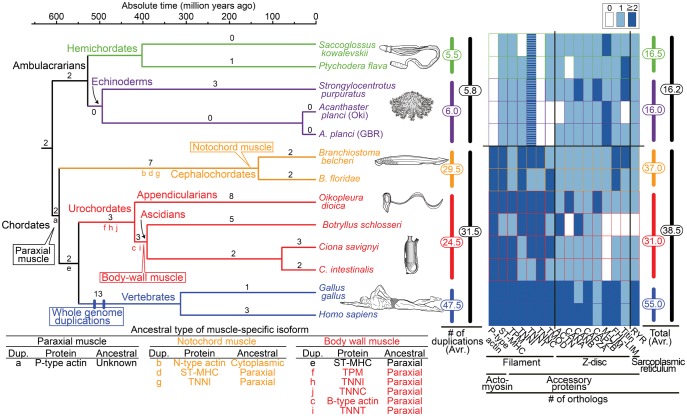
Time-calibrated deuterostome tree (Holland 1996; Simakov et al. 2015) and evolution of structural (contractile protein) genes of paraxial muscle as a summary of gene tree analyses (supplementary fig. S2*A*–*P*, Supplementary Material online). Numbers at branches are counts of isoform families that experienced gene duplication and numbers at vertical bars (middle) are total counts of gene duplications in major lineages (supplementary fig. S3, Supplementary Material online). The heat map (right) reflects the estimated number of orthologs (table 1). Striped boxes in ambulacrarian troponin T indicate ambiguity of their functions as in chordate troponin T. The table (below) indicates inferred ancestral types of muscle-specific isoforms.

#### Troponin I

The troponin complex (fig. 2*A*), in cooperation with tropomyosin, is the thin-filament-associated regulatory switch that controls contractile activation in vertebrate sarcomeric muscle (Schmidt-Rhaesa 2007). Troponin I, the contractile inhibitory component of troponin, is an actin-binding protein that also interacts with the other troponin subunits, troponins T and C. Troponins I and T most likely arose by gene duplication in the bilaterian stem line (Steinmetz et al. 2012). Although the expression and function of *troponin I* are confirmed in sea urchin larval muscle (Yaguchi et al. 2017), troponin I and other components of the troponin complex have not been found in other ambulacrarians (Barnes et al. 2016).

Our analysis identified *troponin I* gene sequences in all five deuterostome lineages (fig. 2*B*4). The existence of troponin T-binding sites and an inhibitory region (supplementary fig. S2*D*4*b*, Supplementary Material online) supports the function of genes included in the Hemichordate-M and Echinoderm-M clades. Cephalochordate genes split into the Cephalochordate-P and -N clades. The sister-group relationship between these two cephalochordate clades suggests that notochord troponin I arose from paraxial troponin I via gene duplication (g in fig. 2*B*4) in the cephalochordate lineage. Urochordate genes formed two clades, Urochordate-PB1 and -PB2, both consisting of genes expressed in paraxial and body-wall muscle (supplementary fig. S2*D*3, Supplementary Material online). Inclusion of body-wall muscle genes within each clade comprising urochordate paraxial genes suggests that body-wall muscle troponin I was derived from paraxial muscle troponin I via gene duplication (h in fig. 2*B*4) in stem urochordates.

#### Troponin T

Our pipeline analysis of chordate *troponin T* genes identified ambulacrarian genes as members of the troponin T orthogroup (fig. 2*B*5), although functions of these ambulacrarian genes were not confirmed due to low conservation of tropomyosin-binding sites (supplementary fig. S2*E*5, Supplementary Material online). Urochordate *troponin T* genes split into two clades, Oikopleura-P and Ascidian-PB/-PA. Inclusion of body-wall muscle genes within both of ascidian clades (supplementary fig. S2*E*4, Supplementary Material online) suggests that body-wall troponin T arose from paraxial troponin T by ascidian-specific gene duplication (i in fig. 2*B*5).

#### Troponin C

The pipeline analysis showed the presence of *troponin C* genes in three chordates and indicated their absence in all ambulacrarians (fig. 2*B*6). Urochordate genes formed two clades, Urochordate-P and -PB. Inclusion of body wall-type genes of several ascidians within one of the duplicated urochordate clades (supplementary fig. S2*F*3, Supplementary Material online) suggests that body wall-type troponin C was derived from paraxial-type via gene duplication (j in fig. 2*B*6) at the base of urochordate divergence.

#### Tropomodulin

Tropomodulin binds to and caps the pointed ends of actin filaments in a tropomyosin-dependent manner and regulates the length of thin filaments by inhibiting association or dissociation from the end (fig. 2*A*). Leiomodin is known only from vertebrates and shares common structural and functional properties with tropomodulin (Colpan et al. 2013).

Our pipeline analysis using *tropomodulin* related genes as query sequences identified clades including genes from the five deuterostome lineages (fig. 2*B*7 and supplementary fig. S2*G*3, Supplementary Material online). In addition to clades consisting of *tropomodulin* genes, Olfactores genes formed a monophyletic group of *leiomodin* genes including Vertebrate-Lmod and Urochordate-Lmod clades. Inclusion of these leiomodin clades within a clade of Olfactores *tropomodulin* genes suggests that leiomodin was derived from tropomodulin via gene duplication (k in fig. 2*B*7) in the stem of the Olfactores.

### Orthologs and Gene Duplication Events

In order to uncover patterns of chordate muscle diversification, orthologs and gene duplication events were counted using 16 estimated gene trees (table 1 and fig. 3). Among the five deuterostome lineages, the number of orthologs was highest in vertebrates (55.0 copies on an average) and lowest in echinoderms (16.0). The number of vertebrate orthologs (55.0) is ∼1.5× higher than in the other two chordate lineages (31.0 in urochordates and 37.0 in cephalochordates). The average number of chordate orthologs (38.5) was >2× larger than that of ambulacrarians (16.2). Even considering the diversification of muscle actins in ambulacrarians by including all *actin* genes (supplementary fig. S2*A*5, Supplementary Material online), by our count, the numbers of all *actin* orthologs of ambulacrarians (5.8) and chordates (7.1) still support the higher diversification of chordate muscle isoforms. When the number of duplications was counted, 5× more events occurred in the chordate lineage (31.5) than in the ambulacrarian lineage (5.8). These results indicate that muscles of chordates were highly diversified in comparison with those of ambulacrarians.

**Table 1.** The Number of Orthologs.

	Actomyosin		Accessory Proteins																
	Filament							Z-Disc								Sarcoplasmic Reticulum			
Gene ID	A	B	C	D	E	F	G	H	I	J	K	L	M	N	O	P	Total	Avr.	
Gene Name^a^	P-Type *Actin*	*ST-MHC*	*TPM*	*TNNI*	*TNNT*	*TNNC*	*TMOD*	*ACTN*	*CANA*	*CANB*	*CAPZA*	*CAPZB*	*Muscle-LIM*	*FHL-LIM*	*Titin*	*RYR*			
*Saccoglossus kowalevskii*	0	1	1	0	3	0	1	1	1	1	1	1	2	1	1	1	16	16.5	16.2
*Ptychodera flava*	0	1	1	1	4	0	1	1	0	1	1	1	1	1	2	1	17		
*Strongylocentrotus purpuratus*	0	1	1	1	4	0	1	1	1	2	1	2	1	1	2	1	20	16.0	
*Acanthaster planci* (OKI)	0	1	1	1	2	0	0	0	1	1	1	1	2	1	0	1	13		
*Acanthaster planci* (GBR)	0	1	1	1	2	0	0	1	1	1	1	1	2	1	1	1	15		
*Branchiostoma belcheri*	6	19	1	2	3	2	1	1	1	1	1	1	1	2	2	1	45	37.0	38.5
*Branchiostoma floridae*	6	5	1	2	1	1	2	1	1	1	1	1	1	3	1	1	29		
*Oikopleura dioica*	3	12	5	4	6	2	3	1	4	1	1	3	1	1	1	1	49	31.0	
*Botryllus schlosseri*	2	1	1	2	3	2	1	2	0	3	1	1	0	0	0	0	19		
*Ciona savignyi*	6	8	3	1	2	2	2	1	1	1	1	1	0	1	1	1	32		
*Ciona intestinalis*	5	3	3	1	1	2	1	1	1	1	1	1	0	1	1	1	24		
*Gallus gallus*	4	11	4	2	2	2	7	3	2	1	3	1	3	4	1	2	52	55.0	
*Homo sapiens*	4	10	4	3	3	2	7	4	3	3	3	1	3	4	1	3	58		

a P-type actin, paraxial type *actin* (supplementary fig. S2*A*, Supplementary Material online); *ST-MHC, myosin heavy chain, skeletal* (supplementary fig. S2*B*, Supplementary Material online); *TPM, tropomyosin* (supplementary fig. S2*C*, Supplementary Material online); *TNNI, troponin I* (supplementary fig. S2*D*, Supplementary Material online); *TNNT, troponin T* (supplementary fig. S2*E*, Supplementary Material online); *TNNC, troponin C* (supplementary fig. S2*F*, Supplementary Material online); *TMOD, tropomodulin* (supplementary fig. S2*G*, Supplementary Material online); *ACTN, alpha-actinin* (supplementary fig. S2*H*, Supplementary Material online); *CANA, calcineurin A* (supplementary fig. S2*I*, Supplementary Material online); *CANB*, *Calcineurin B* (supplementary fig. S2*J*, Supplementary Material online); *CAPZA, capping protein, alpha* (supplementary fig. S2*K*, Supplementary Material online); *CAPZB, capping protein, beta* (supplementary fig. S2*L*, Supplementary Material online); *Muscle-LIM* (supplementary fig. S2*M*, Supplementary Material online); *FHL-LIM, four and a half LIM domains* (supplementary fig. S2*N*, Supplementary Material online); *Titin* (supplementary fig. S2*O*, Supplementary Material online); *RYR*, *ryanodine receptor, skeletal* (supplementary fig. S2*P*, Supplementary Material online).

## Discussion

### Origin of Paraxial Striated Muscle

The present study demonstrated that among the 16 muscle protein isoform families with gene trees that fulfilled our BS 70% criterion, paraxial muscle *actin* and *troponin C* orthologs are not present in ambulacrarian genomes (fig. 3 right). Although our pipeline analysis of muscle actin (fig. 2*B*1) did not clearly identify gene duplications that produced paraxial muscle actin due to the highly conserved sequences of cytoplasmic and muscle *actin* genes among nonchordates, the monophyletic origin of chordate paraxial muscle actin (Chordate-P) was supported by a high bootstrap value (supplementary fig. S2*A*5, Supplementary Material online). Considering the presence of the *troponin C* gene in almost all reported bilaterians, the estimated gene tree of troponin C (fig. 2*B*6) suggests that troponin C was present in the bilaterian ancestor and has been lost in the ambulacrarian ancestor, as suggested previously (Barnes et al. 2016; Yaguchi et al. 2017). Thus, our results suggest that by acquiring paraxial actin as a fast “rail” muscle and preserving troponin C as an essential mediator of fast constriction, the chordate ancestor formed paraxial striated muscle to evolve a free-swimming lifestyle.

Despite the absence of paraxial *actin* and *troponin C* genes, our analyses indicate that homologs of the other 14 muscle genes are present in ambulacrarians (fig. 3 right). Given that expression and functions of *Drosophila* or *C. elegans* homologs, those of ambulacrarian homologs are considered to be conserved among the 14 genes, although tropomyosin-binding sites were not conserved in *troponin T* genes. In fact, expression of *ST-MHC* (SPU010054 in supplementary fig. S2*B*, Supplementary Material online), *tropomyosin* (SPU000128 in supplementary fig. S2*C*, Supplementary Material online, Andrikou et al. 2013), and *troponin I* (LC187281 in supplementary fig. S2*D*, Supplementary Material online, Yaguchi et al. 2017) genes was observed in circumesophageal muscle of sea urchin, and the function of tropomyosin gene (Sakowv30041361 in supplementary fig. S2*C*, Supplementary Material online) is suggested in acorn worm muscle via biochemical analysis (Sonobe et al. 2016).

The absence of the *troponin C* gene in ambulacrarian genomes reflects differences in regulating mechanisms of muscle contraction between chordates and ambulacrarians. In most striated muscles, troponin C generally functions as the Ca^2+^ sensor of the troponin complex and helps trigger muscle contraction (Farah and Reinach 1995). For troponin–tropomyosin regulation in ambulacrarian muscle, Yaguchi et al. (2017) proposed two alternative hypotheses. The first is that the complex, consisting of two troponin components, troponin I and T, functions independently. The second is that calmodulin (Jensen et al. 2015) mediates regulation as an alternative to troponin C, given the sequence similarity between those two genes. In fact, our analysis suggests the presence of *calmodulin* orthologs among all analyzed ambulacrarian genomes (supplementary fig. S2*R*, Supplementary Material online).

### Origin of Cephalochordate Notochord Striated Muscle

Some structural details (Flood 1975), as well as expression of the cephalochordate *Brachyury* gene in the notochord (Holland et al. 1995), suggest that cephalochordate and vertebrate notochords are homologous. However, the role of the notochord in cephalochordates is quite different from that in vertebrate embryos, which functions as a source of signals required for body plan formation (Satoh 2016). Due to the presence of striated muscle and its mechanical properties upon nervous stimulation, the notochord of adult cephalochordates is considered a mechanical swimming organ (Guthrie and Banks 1970). Given the basal placement of cephalochordates among chordates, the occurrence of notochord muscle in cephalochordates may be explained by one of two evolutionary scenarios: 1) acquisition in the cephalochordate lineage or 2) acquisition before the chordate divergence with losses in the urochordate and vertebrate lineages (Lauri et al. 2014). Owing to the absence of notochord muscle cells in all urochordates and vertebrates, the origin of cephalochordate notochord muscle could not be examined further by morphological analysis.

We surmised the origin of notochord muscle using resultant trees of the 16 muscle genes. Among analyses of paraxial *actin*, *ST-MHC*, and *troponin I* genes (fig. 3 bottom) including genes expressed in cephalochordate notochord, estimated ancestral types of notochord isoforms were different. Notochord isoforms of ST-MHC and troponin I were derived from paraxial forms, whereas notochord actin came from cytoplasmic actin. In contrast, phylogenetic positions of gene duplications producing the three notochord isoforms were consistently placed in the stem cephalochordate lineage (b, d, and g in fig. 3). Given that cephalochordate genes of a transcription factor of notochord-type actin, MyoD (Urano et al. 2003), were derived from tandem gene duplications in the cephalochordate lineage (supplementary fig. S2*S*, Supplementary Material online), the present study suggests that notochord muscle was formed in the cephalochordate lineage, but not before the chordate divergence, despite the cytoplasmic origin of notochord actin.

### Origin of Ascidian Body-Wall Smooth Muscle

Body-wall smooth muscle shared among adult ascidians also presents greater difficulties in terms of recognizing homologous muscles (fig. 1). However, as for troponin–tropomyosin regulation (Toyota et al. 1979; Ohshima et al. 1988) and expression of a MyoD-like transcription factor (Araki et al. 1994; Meedel et al. 1997), muscle of ascidian body wall most resembles vertebrate skeletal muscle. Among the six protein isoform families analyzed (fig. 3 bottom), all six body-wall isoforms were consistently derived from paraxial-type. In contrast, phylogenetic positions of gene duplications producing the six body-wall muscle isoforms were slightly different. Although the duplication producing body wall-type ST-MHC (e in fig. 3) was placed before the diversification of urochordates and vertebrates, as shown in Chiba et al. (2003), those producing the other five body wall-type isoforms occurred within the urochordate lineage (c and i in stem ascidians and f, h, and j in stem urochordates).

These results indicate that most isoforms of notochord or body-wall muscles in nonvertebrate chordates arose from paraxial muscle (fig. 3). Gene duplications producing specialized isoforms occurred at the same time or just before emergence of the two lineage-specific muscles, not before the rise of chordates.

### Timing of Filament and Z-Disc Diversification

The present study suggests that in chordate muscles, diversification of contractile proteins is highest in muscle filaments, especially in actomyosin (table 1 and fig. 3 right). Among orthologs of seven genes expressed in vertebrate filaments, copy numbers were larger in chordates (most have more than two copies) than in ambulacrarians (0 or a single copy, except for troponin T). The increasing number of isoforms expressed in filaments may have played an important role in enabling fast and variable contraction of paraxial muscle.

Z-discs, the lateral borders of sarcomeres, hold actin filaments in place and anchor myosin filaments using an elastic protein called titin (fig. 2*A*). In soft-bodied animals without Z-discs, cross-striated muscles with aligned Z-elements appear to occur in connection with fast movement in a defined, predictable range (Schmidt-Rhaesa 2007). Thus, the presence of Z-discs among the three chordate lineages suggests that this structure was present in paraxial muscle when the chordate ancestor started swimming with its tail.

In the present study, orthologs of all eight genes expressed in vertebrate Z-discs (e.g., *alpha-actin* and *CAPZA* in fig. 3 right) were found in ambulacrarians. Although ambulacrarian orthologs without protostome orthologs could not be evaluated for five Z-disc proteins (e.g., myopalladin and myotillin in supplementary table S2, Supplementary Material online) due to the tenuous nature of sequence alignments, the present study suggests that ambulacrarians do not form Z-discs, in spite of the presence of these eight proteins.

Although copy numbers of orthologs of Z-disc genes were similar between ambulacrarians and nonvertebrate chordates, they differed among the three chordate lineages (fig. 3 right). While most vertebrate orthologs were present in more than two copies, cephalochordate and urochordate orthologs were present in a single copy, as in ambulacrarians. The smaller numbers of cephalochordate and urochordate orthologs observed are compatible with simple Z-disc structures in these lineages compared with those of vertebrates (Burighel and Cloney 1997).

### Timing of Gene Duplications

Gene duplications in peripheral branches of deuterostomes (supplementary fig. S3 right, Supplementary Material online) (16.3 copies on an average) were three times more numerous than in ancestral branches (5.3). This indicates that most gene duplications leading to muscle isoform diversification occurred within deuterostome lineages.

Most orthologs of the vertebrate lineage (55.0 in fig. 3 right) were derived from gene duplications in the stem vertebrate, probably during whole genome duplication events (Holland 2013). In addition, orthologs of the Oikopleura (49 in table 1) and cephalochordate (37.0) lineages were mainly derived from gene duplications in the stem Oikopleura and cephalochordates, respectively. The large numbers of orthologs observed in cephalochordate, Oikopleura, and vertebrate lineages are compatible with their use of paraxial striated muscles during larval and adult stages (fig. 1). Occurrences of many duplications at stem nodes reflect the long history of developed paraxial striated muscles within each lineage.

## Conclusions

It has been suggested that retained paralogs derived from whole genome duplications enabled the emergence of vertebrate-specific structures such as the neural crest, the midbrain/hindbrain organizer, and neurogenic placodes (Holland 2013). In fact, the present study suggested that 13 out of 16 analyzed muscle genes were duplicated at the base of vertebrates, probably as vestiges of whole genome duplications. In contrast, large numbers of gene duplications were not identified in the stem line of chordates by analyses of genes related to muscle (this study) or to notochord formation (Inoue et al. 2017). Therefore, it is less likely that duplications played an important role at least in the emergence of paraxial muscle, although lineage-specific gene duplications produced most isoforms of muscle contractile proteins. For future studies exploring the evolutionary origin of paraxial striated muscle, in addition to expression patterns of related genes and functions of paraxial actin and troponin C, regulatory mechanisms of muscle genes should be considered.

## Materials and Methods

Gene trees were estimated using an analytical pipeline (supplementary fig. S1, Supplementary Material online) implementing BLAST searches and the maximum likelihood method (modified from Inoue et al. 2015, 2017).

### BLAST Search

Protein-coding sequences (amino acids) from humans and other deuterostomes were used as queries for BLASTP searches (Altschul et al. 1997) (supplementary fig. S1*A*1, Supplementary Material online) against all protein-coding sequences in 16 selected animal genomes (supplementary table S1, Supplementary Material online). The top BLAST hits were screened using an E-value cutoff of <10^−3^. Where transcript variants existed for a single locus, only the longest sequence was used in the present analysis.

### Alignment

Primary sequences of proteins identified by BLASTP searches were aligned using MAFFT (Katoh et al. 2005). Multiple sequence alignments were trimmed by removing poorly aligned regions using TRIMAL 1.2 (Capella-Gutierrez et al. 2009) with the option “gappyout.” Corresponding cDNA sequences were forced onto the amino acid alignment using PAL2NAL (Suyama et al. 2006) to generate nucleotide alignments for later comparative analysis. Each gene sequence was checked and removed from the alignment if it was shorter than 55% of the length of the query sequence at unambiguously aligned sites (supplementary fig. S1*A*2, Supplementary Material online).

### Gene Tree Search

Gene trees were estimated using neighbor-joining (NJ, supplementary fig. S1*A*3, Supplementary Material online) and maximum likelihood (ML, supplementary fig. S1*B*5, Supplementary Material online) methods, with codon positions of each sequence aligned by bootstrap analysis based upon 100 replicates. The analyses were conducted using data sets that comprised nucleotide sequences without 3rd codon positions (Exc3rd). For analyses based upon short query sequences or analyses within specific lineages (supplementary fig. S1*B*5’, Supplementary Material online), data sets including 3rd codon positions (Inc3rd) were used. Amino acid sequences were not used for phylogenetic analyses due to low resolution probably caused by the short data set (Inoue et al. 2017). To select ambulacrarian/chordate ortholog candidates from BLASTP hit sequences, NJ analysis was conducted using the software package Ape in R with the TN93 model (Tamura and Nei 1993).

Resultant gene trees, however, often have some weakly supported nodes. In such cases, one needs to revise ambiguous nodes in comparison with the topology of broadly accepted phylogenetic relationships—the species tree. For this purpose, we then conducted rearrangement analysis using a method implemented in NOTUNG (Chen et al. 2000) for the NJ gene tree compared with the species tree (supplementary fig. S1*A*4, Supplementary Material online). As a first step, NOTUNG rearranges weakly supported nodes of the gene tree to minimize duplication and extinction of genes, using parsimony with equal weights. We set the threshold to 70% for bootstrap support values of nodes. Then, the rearranged tree was reconciled with the species tree.

To select reliable orthogroups, NJ trees derived from rearrangement analysis were filtered using the BS 70% criterion based upon the bootstrap value of a key node, separation of protostomes and deuterostomes (supplementary fig. S1*A*, Supplementary Material online). For a cutoff value, we set the bootstrap probability at 70% for the key node to avoid including orthogroups identified with ambiguous gene trees.

To estimate gene duplication events, selected orthologs from the rearranged NJ tree were realigned and subjected to codon-partitioned ML analysis (supplementary fig. S1*B*5, Supplementary Material online). The analysis was performed with RAxML 8.2.4 (Stamatakis 2014), which invokes a rapid bootstrap analysis and searches for the best-scoring ML tree with the GTRGAMMA (general time-reversible with the gamma) (Yang 1994a, 1994b) model. To implement constraints on some monophyletic groups within a lineage, selected orthologs of focal lineages were realigned and subjected to ML analysis (supplementary fig. S1*B*5’, Supplementary Material online). Identified monophyletic groups with >70% bootstrap values were used as constraints using the option “-g” in RAxML for subsequent analysis of deuterostome relationships. Resulting ML trees were subjected to rearrangement analysis (supplementary fig. S1*B*6, Supplementary Material online).

## Supplementary Material

Supplementary data are available at *Molecular Biology and Evolution* online.

## Supplementary Material

Supplementary Data
