# Supplementary material for: Deuterostome Genomics: Lineage-Specific Protein Expansions That Enabled Chordate Muscle Evolution
Source: Mol Biol Evol. 2018 Jan 8;35(4):914–24. doi: 10.1093/molbev/msy002 (PMC5888912; doi:10.1093/molbev/msy002)
Supplement: Supplementary Data [file msy002_supp.zip › msy002_supp_inoue.pdf]

Supplementary materials

(Inoue et al)

Table S1. A list of bilaterian species with decoded genomes used in this study.

Table S2. Results of gene tree analyses of muscle proteins.

FIG. S1. Schematic outline of the phylogeny-based pipeline used to identify deuterostome orthogroups and internal relationships. The species tree is based on Simakov et al. (2015) and Holland (1996). As discussed in Holland (1996), no estimated muscle gene tree (Fig. S2) supported the hypothesis including appendicularians in ascidians (dashed line).

FIG. S2. Gene trees of muscle proteins.

(A) Actin.

1. Deuterostome tree (Exc3rd dataset, 750 sites).

Numbers beside nodes indicate bootstrap probabilities (> 50%). Query sequences used for BLAST searches are marked with black (reference for spurious blast hits removal [Fig. S1A2]) or gray dots. In addition to the phylogenetic analysis, expression sites were evaluated on the basis of diagnostic amino acid sites (Suzuki and Satoh 2000; Chiba et al. 2003). Identities to one of the query gene sequences are denoted by dots. The Cephalochordate-N clade was deeply nested within cytoplasmic-type sequences (Cephalochordate-C).

2. Chordate tree (Inc3rd dataset, 1125 sites).

Nodes marked with gray dots were used as constraints in the subsequent ML analysis subjected to rearrangement analysis (Fig. S1B5).

3. Vertebrate tree (Inc3rd dataset, 1128 sites).

4. Ascidian tree (Inc3rd dataset, 1134 sites).

5. Rearranged gene tree (Exc3rd dataset).

Gene sequences expression expected in paraxial or notochord muscle are connected by thick branches. According to the results of local analyses (2, 3, and 4), monophyletic

relationships are constrained for nodes marked with white dots. Rearranged nodes are indicated with ‘r’ and gene duplication events are marked by ‘D’ in accordance with the result of rearrangement analysis (Fig. S1B6). Gene duplications marked with black vertical bars are discussed in the main text.

(B) Myosin heavy chain, skeletal (ST-MHC).
1. Deuterostome tree (Exc3rd dataset, 3808 sites).
2. Olfactores tree (Inc3rd dataset, 5742 sites). Phylogenetic relationships among vertebrate *ST-MHC* genes should be confirmed by denser taxonomic sampling in the future.
3. Rearranged gene tree (Exc3rd dataset).

(C) Tropomyosin (TPM).
Unexpectedly longer sequences (>1500 residues: BRAFL286785 and BRBE011280R) were removed from the analyses.
1. Deuterostome tree (Exc3rd dataset, 568 sites).
2. Olfactores tree (Inc3rd dataset, 843 sites).
3. Rearranged gene tree (Exc3rd dataset).
4. Amino acid sequence alignment of *tropomyosin* genes.
Functions of three sequences in the Echinoderm-C/M clade (underlined) were confirmed by the existence of troponin T binding sites in N terminal (a) and C terminal (b) regions.

(D) Troponin I (TNNI).
1. Deuterostome tree (Inc3rd dataset, 390 sites).
2. Chordate tree (Inc3rd dataset, 426 sites).
3. Rearranged gene tree (Inc3rd dataset).
4. Amino acid sequence alignment of *troponin I* genes.
Previous studies suggested that *Halocynthia* has distinct larval and adult *troponin I* genes (Yuasa et al. 1997; 2002) whereas *Ciona* does not have a specialized larval *troponin I* and the same gene is expressed in both adult (heart and body-wall muscle) and embryonic/larval tail muscle cells (Chiba et al. 2003).

(a) N terminal region.

Two cephalochordate sequences (BRAFL124626 and BRBE261920R-t1, underlined) in the Cephalochordate-P clade were identified as paraxial-type due to the extension of the N-terminal region in comparison with those of notochord-type (Cephalochordate-N clade).

(b) Troponin T binding region.

Functions of gene sequences (underlined) in the Hemichordate-M (PF10037-t) and Echinoderm-M (oki41-31, gbr182-36, and SPU013183-tr) clades were confirmed by the existence of troponin-T binding sites (blue) and an inhibitory region (gray) (Gomes et al. 2002; Patchell et al. 2002).

(c) C-terminal region.

One of the *Botryllus* gene sequences (botctg059511g21644, underlined) was confirmed as a larval-type due to truncation of a C-terminal-type seen in *Halocynthia* larval-type (Cleto et al. 2003), in addition to the phylogenetic position (Pleurogonan-P2 clade in Fig. S2D3). Then the other *Botryllus* sequence (botctg006897g52162) was identified as adult-type.

(E) Troponin T (TNNT).

An unexpectedly long sequence (BRBE023460F-t1, 4501 residues) was removed from the analysis.

1. Deuterostome tree (Exc3rd dataset, 540 sites).
2. Ambulacrarian tree (Exc3rd dataset, 458 site).
3. Olfactores tree (Inc3rd dataset, 543 site).
4. Rearranged gene tree (Exc3rd dataset).

Previous studies suggested that two *troponin T* gene sequences are present in *Halocynthia* (Endo et al. 1996) and *Botryllus* (Degasperi et al. 2009), one larval and the other expressed in adult body-wall muscle, but a single gene with alternatively spliced isoforms is present in *Ciona* (Chiba et al. 2003).

5. Amino acid sequence alignment of *troponin T* genes.

Functions of ambulacrarian genes, like those of chordate genes, were not confirmed due to the low preservation of tropomyosin-binding sites 1 and 2 (Jin and Chong 2010).

(F) Troponin C (TNNC).

The Drosophila *troponin C* gene sequence was added manually.

1. Deuterostome tree (Inc3rd dataset, 456 sites).
2. Olfactores tree (Inc3rd dataset, 507 sites).
3. Rearranged gene tree (Inc3rd dataset).

Despite lower bootstrap values (<70% in the deuterostome tree), monophyly of urochordate or vertebrate genes (nodes marked with white dots and broken-lines) was constrained due to the existence of these clades in the above two analyses.

(G) Tropomodulin (TMOD).

1. Deuterostome tree (Exc3rd dataset, 704 sites).
2. Olfactores tree (Inc3rd dataset, 903 sites).
3. Rearranged gene tree (Exc3rd dataset).

(H) Alpha-actinin (ACTN) rearranged gene tree.

Alpha-actinin cross-links parallel actin filaments (Fig. 2A).

(I) Calcineurin A (CANA) rearranged gene tree.

Calcineurin is present at the M line and may mediate dephosphorylation events of M-line proteins (Hu et al. 2015). Calcineurin A is also known as protein phosphatase 3 catalytic subunit alpha (PPP3CA).

(J) Calcineurin B (CANB) rearranged gene tree.

(K) Capping protein muscle Z-disc, alpha (CAPZA) rearranged gene tree.

CAPZ (Fig. 2A) caps the barbed ends of actin filaments and interacts strongly with α-actinin and nebulin (Luther 2009).

(L) Capping protein muscle Z-disc, beta (CAPZB) rearranged tree.

(M) Muscle-LIM rearranged gene tree.

Muscle LIM protein localizes to the sarcomeric Z-disc, where it most likely interacts

with α-actinin, calcineurin, and telethonin (Knoll et al. 2011). Muscle-LIM protein is also known as cysteine and glycine-rich protein (CSRP).

(N) Four-and-a-half-LIM-domains (FHL-LIM) rearranged gene tree.
FHL-LIM is enriched in mammalian striated muscle and associated with multiple binding partners and biological functions, including striated muscle development and function (Sheikh et al. 2008).

(O) Titin rearranged gene tree.
Titin (connectin) is the largest protein that connects the Z-disc to the M-line of the sarcomere in heart and skeletal muscle (Fig. 2A) and maintains the sarcomere during muscle contraction and relaxation.

(P) Ryanodine receptor, skeletal (RYR) rearranged gene tree.
Ryanodine receptors are located in the sarcoplasmic/endoplasmic reticulum membrane and are responsible for the release of Ca^{2+} from intracellular stores during excitation-contraction coupling in both cardiac and skeletal muscle (Lanner et al. 2010).

(Q) Myosin heavy chain, smooth (SM-MHC) rearranged gene tree.
The estimated gene tree indicates that vertebrate smooth muscle-type of MHC was derived from SM-MHC-type via gene duplication occurred in stem vertebrates, as shown in (Kusakabe et al. 2004).

(R) Calmodulin (CALM) rearranged gene tree.
Calmodulin, the ubiquitous and multifunctional Ca^{2+}-binding protein, mediates many of the regulatory effects of Ca^{2+}, including the contractile state of smooth muscle (Walsh 1994).

(S) Myogenic differentiation (MyoD) ML tree.
MyoD, a member of the myogenic regulatory factor (MRF) family, directly controls contractile genes in skeletal (and esophageal) striated myocytes, cooperatively with MEF2, but is absent from smooth and cardiac muscles (Brunet et al. 2016).

(T) Serum response factor (SRF) ML tree.

SRF interacts with myogenic regulatory factors, MyoD, MYF5, myogenin, and MRF4 to regulate skeletal muscle gene expression (Meadows et al. 2008).

(U) Myocyte enhancer factor 2 (MEF2) ML tree.

MEF2, a member of the MADS-box super family of transcriptional regulatory proteins, is an established component in regulation of a diverse number of tissues, including skeletal, cardiac, and smooth muscle, neurons, and T cells (Wales et al. 2014).

Fig. S3. Time-calibrated tree of deuterostomes and gene duplication events of muscle proteins.

References

Cleto CL, Vandenberghe AE, MacLean DW, Pannunzio P, Tortorelli C, Meedel TH, Satou Y, Satoh N, Hastings KE. 2003. Ascidian larva reveals ancient origin of vertebrate-skeletal-muscle troponin I characteristics in chordate locomotory muscle. *Mol Biol Evol* 20:2113–2122.

Degasperi V, Gasparini F, Shimeld SM, Sinigaglia C, Burighel P, Manni L. 2009. Muscle differentiation in a colonial ascidian: organisation, gene expression and evolutionary considerations. *BMC Dev Biol* 9:48.

Endo T, Matsumoto K, Hama T, Ohtsuka Y, Katsura G, Obinata T. 1996. Distinct troponin T genes are expressed in embryonic/larval tail striated muscle and adult body wall smooth muscle of ascidian. *J Biol Chem* 271:27855–27862.

Gomes AV, Potter JD, Szczesna-Cordary D. 2002. The role of troponins in muscle contraction. *IUBMB Life* 54:323–333.

Hu LY, Ackermann MA, Kontrogianni-Konstantopoulos A. 2015. The sarcomeric M-region: a molecular command center for diverse cellular processes. *Biomed Res Int* 2015:714197.

Jin JP, Chong SM. 2010. Localization of the two tropomyosin-binding sites of troponin T. *Arch Biochem Biophys* 500:144–150.

Knoll R, Buyandelger B, Lab M. 2011. The sarcomeric Z-disc and Z-discopathies. *J Biomed Biotechnol* 2011:569628.

Kusakabe R, Takechi M, Tochinai S, Kuratani S. 2004. Lamprey contractile protein genes mark different populations of skeletal muscles during development. *J Exp Zool B Mol Dev Evol* 302:121–133.

Lanner JT, Georgiou DK, Joshi AD, Hamilton SL. 2010. Ryanodine receptors: structure, expression, molecular details, and function in calcium release. *Cold Spring Harb Perspect Biol* 2:a003996.

Luther PK. 2009. The vertebrate muscle Z-disc: sarcomere anchor for structure and signalling. *J Muscle Res Cell Motil* 30:171–185.

Meadows SM, Warkman AS, Salanga MC, Small EM, Krieg PA. 2008. The myocardin-related transcription factor, MASTR, cooperates with MyoD to activate skeletal muscle gene expression. *Proc Natl Acad Sci U S A* 105:1545–1550.

Patchell VB, Gallon CE, Hodgkin MA, Fattoum A, Perry SV, Levine BA. 2002. The inhibitory region of troponin-I alters the ability of F-actin to interact with different segments of myosin. *Eur J Biochem* 269:5088–5100.

Sheikh F, Raskin A, Chu PH, Lange S, Domenighetti AA, Zheng M, Liang X, Zhang T, Yajima T, Gu Y, et

al. 2008. An FHL1-containing complex within the cardiomyocyte sarcomere mediates hypertrophic biomechanical stress responses in mice. *J Clin Invest* 118:3870–3880.
Wales S, Hashemi S, Blais A, McDermott JC. 2014. Global MEF2 target gene analysis in cardiac and skeletal muscle reveals novel regulation of DUSP6 by p38MAPK-MEF2 signaling. *Nucleic Acids Res* 42:11349–11362.
Walsh MP. 1994. Calmodulin and the regulation of smooth-muscle contraction. *Mol Cell Biochem* 135:21–41.
Yuasa HJ, Kawamura K, Yamamoto H, Takagi T. 2002. The structural organization of ascidian *Halocynthia roretzi* troponin I genes. *J Biochem* 132:135–141.
Yuasa HJ, Sato S, Yamamoto H, Takagi T. 1997. Primary structure of troponin I isoforms from the ascidian *Halocynthia roretzi*. *J Biochem* 122:374–380.

Table S1. A list of bilaterian species with decoded genomes used in this study.

Species	# of genes
Protostomia	
Ecdysozoa	
Caenorhabditis elegans[a]	20,447
Drosophila melanogaster[a]	13,918
Deuterostomia	
Ambulacraria	
Hemichordata	
Saccoglossus kowalevskii[b]	34,239
Ptychodera flava[b]	34,647
Echinodermata	
Strongylocentrotus purpuratus[a]	28,842
Acanthaster planci (Okinawa)[c]	24,323
Acanthaster planci (Great Barrier Reef)[c]	24,747
Chordata	
Cephalochordata	
Branchiostoma belcheri[d]	21,954
Branchiostoma floridae[e]	30,392
Urochordata	
Appendicularia	
Oikopleura dioica[f]	17,212
Ascidiacea	
Botryllus schlosseri[g]	30,910
Ciona savignyi[h]	11,616
Ciona intestinalis[h,i]	16,671
Vertebrata	
Gallus gallus[h]	15,508
Homo sapiens[h]	22,489

[a] EnsemblMetazoa33. Ensembl, Hinxton. 2016. http://metazoa.ensembl.org/index.html. Accessed 23 Apr 2016.
[b] Hemichordate Genomes. Okinawa Institute of Science and Technology Graduate University, Okinawa. 2016. https://groups.oist.jp/molgenu/hemichordate-genomes. Accessed 7 Apr 2016.
[c] MarinegenomicsDB. Okinawa Institute of Science and Technology Graduate University, Okinawa. 2013. http://marinegenomics.oist.jp/gallery. Accessed 25 Apr 2016.
[d] LanceletDB, v18h27r3. Sun Yatsen University, Guangzho. 2014. http://genome.bucm.edu.cn/lancelet/gbrowser_wel.php. Accessed 15 Jul 2016.
[e] Branchiostoma floridae-JGI Genome Portal, v1. Joint Genome Institute, Walnut Creek. 2014. http://genome.jgi.doe.gov/Brafl1/Brafl1.home.html. Accessed 15 Jul 2016.
[f] OikoBase. University of Bergen, Bergen. 2013. http://oikoarrays.biology.uiowa.edu/Oiko. Accessed 23 Apr 2016.
[g] Botryllus schlosseri Genome Project. Stanford University, Stanford. 2013. http://botryllus.stanford.edu/botryllusgenome. Accessed 24 Apr 2016.
[h] Ensembl79. Ensembl, Hinxton. 2015. http://mar2015.archive.ensembl.org/index.html. Accessed 5 May 2015.
[i] *C. intestinalis*2: ANISEED, C.intestinalis, Old Transcript Models, Kyoto Grail. 2005. https://www.aniseed.cnrs.fr/aniseed/download/download_data. Accessed 11 May 2016.

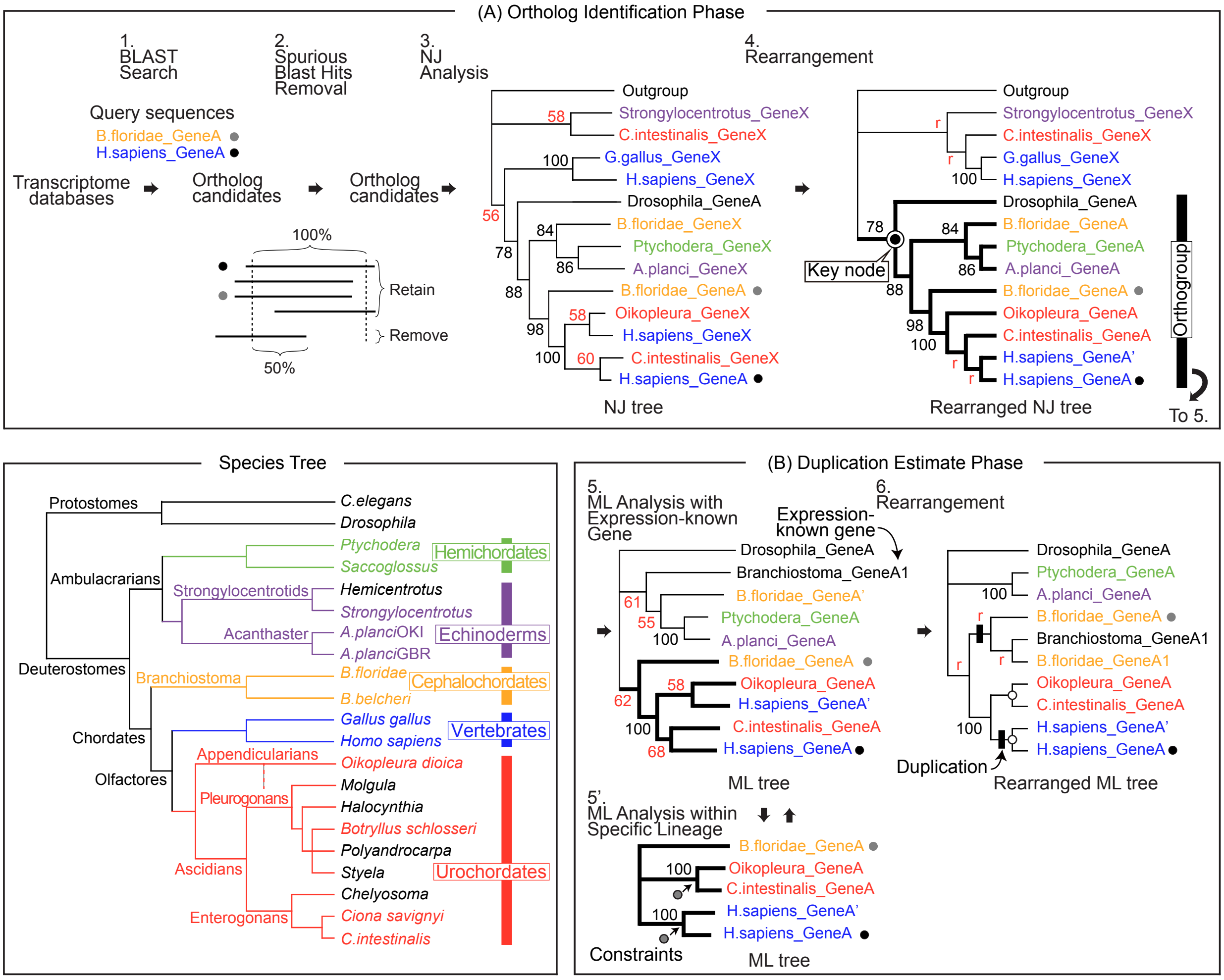
(A) Ortholog Identification Phase
1. BLAST Search
2. Spurious Blast Hits Removal
3. NJ Analysis
4. Rearrangement
Query sequences
B.floridae_GeneA
H.sapiens_GeneA
Transcriptome databases
Ortholog candidates
Ortholog candidates
100%
Retain
Remove
50%
NJ tree
Rearranged NJ tree
Key node
Orthogroup
To 5.
Species Tree
Protostomes
Ambulacrarians
Deuterostomes
Chordates
Olfactores
Hemichordates
Echinoderms
Cephalochordates
Vertebrates
Urochordates
(B) Duplication Estimate Phase
5. ML Analysis with Expression-known Gene
6. Rearrangement
Expression-known gene
ML tree
Duplication
Rearranged ML tree
5'. ML Analysis within Specific Lineage
Constraints
ML tree

Fig. S1

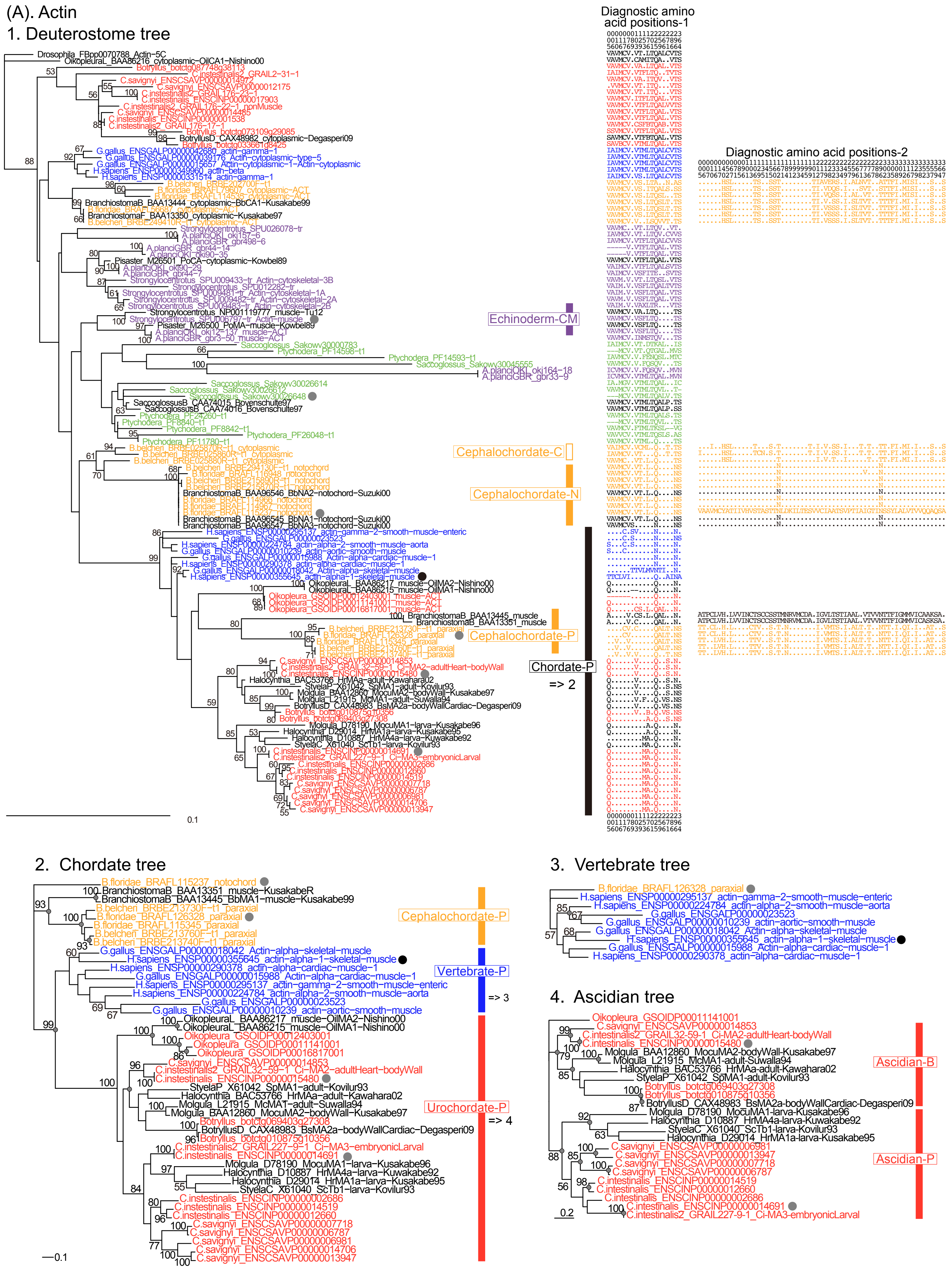

Fig. S2A
(continued)

5. Rearranged gene tree

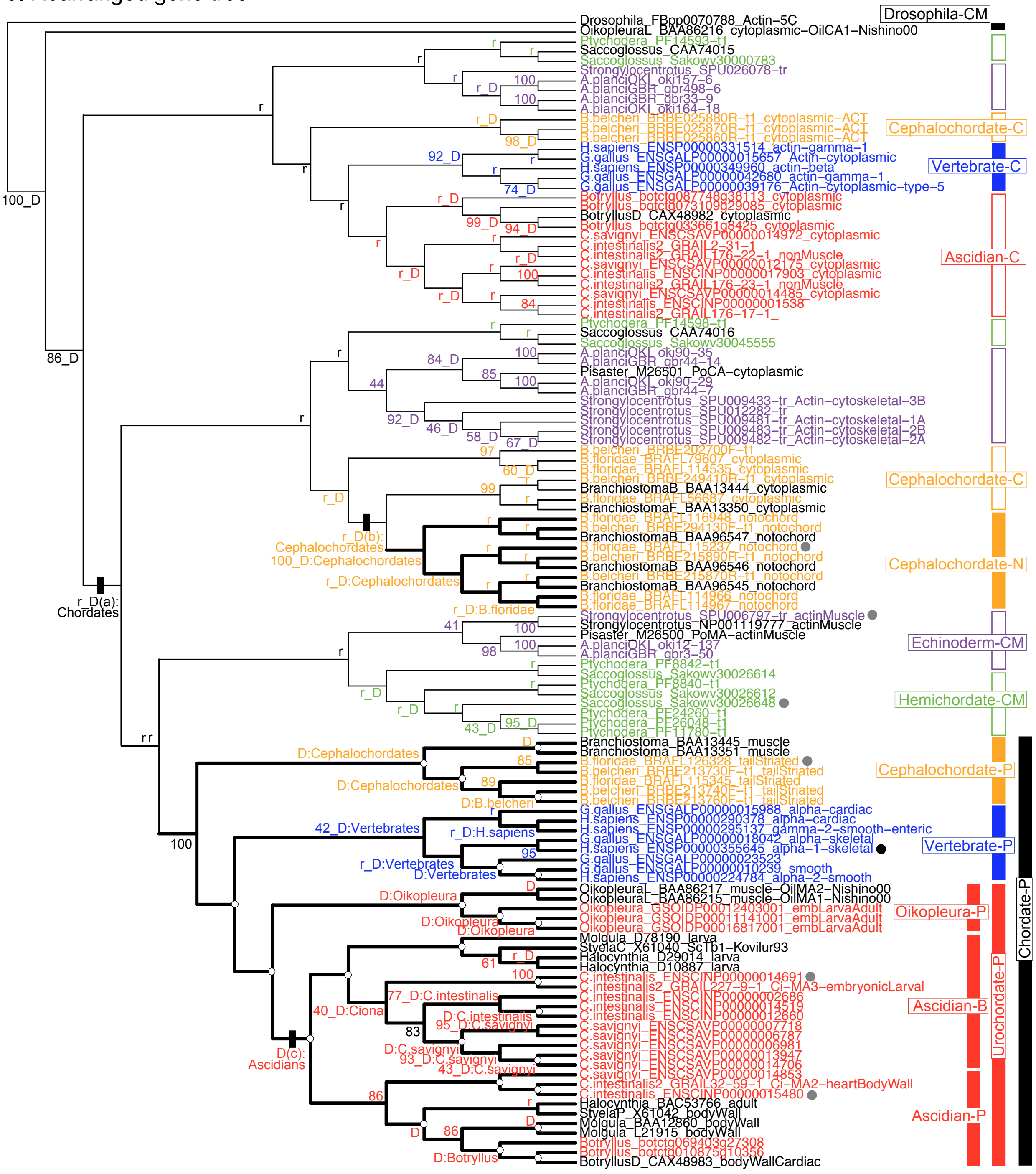

Fig. S2A
(continued)

(B) ST-MHC

1. Deuterostome tree

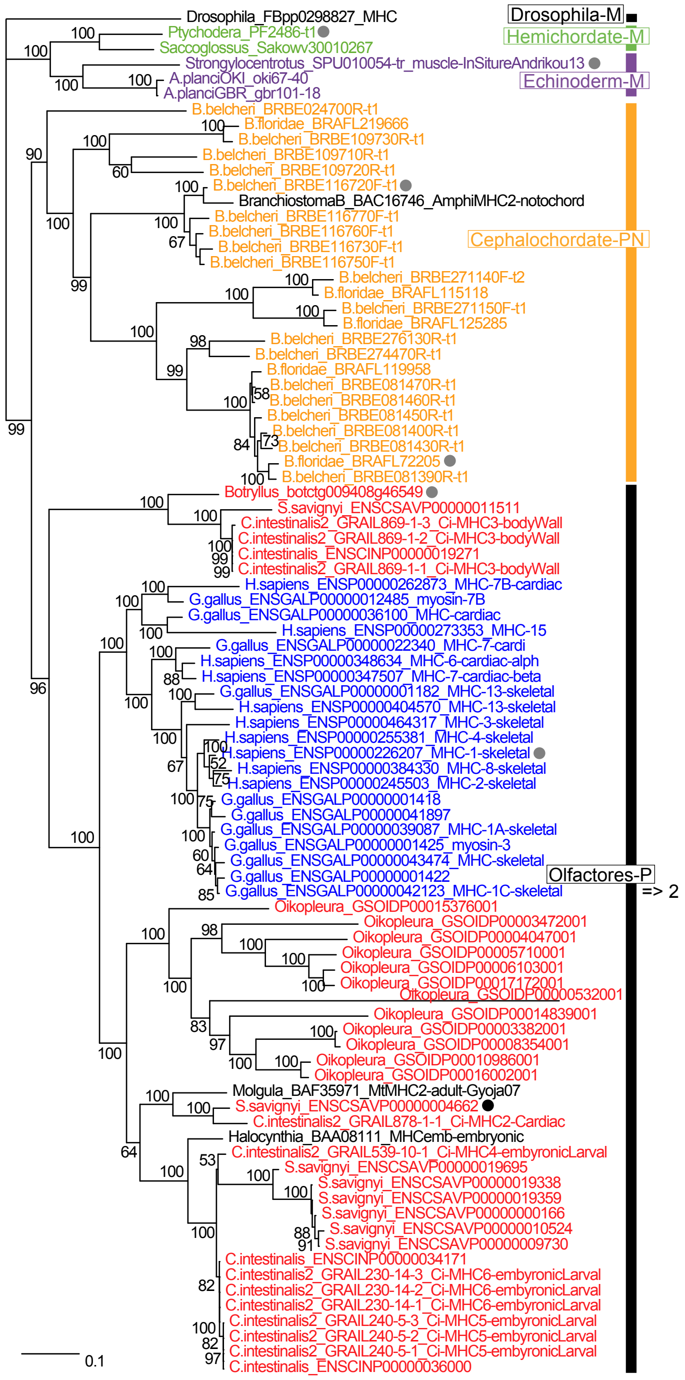

2. Olfactores tree

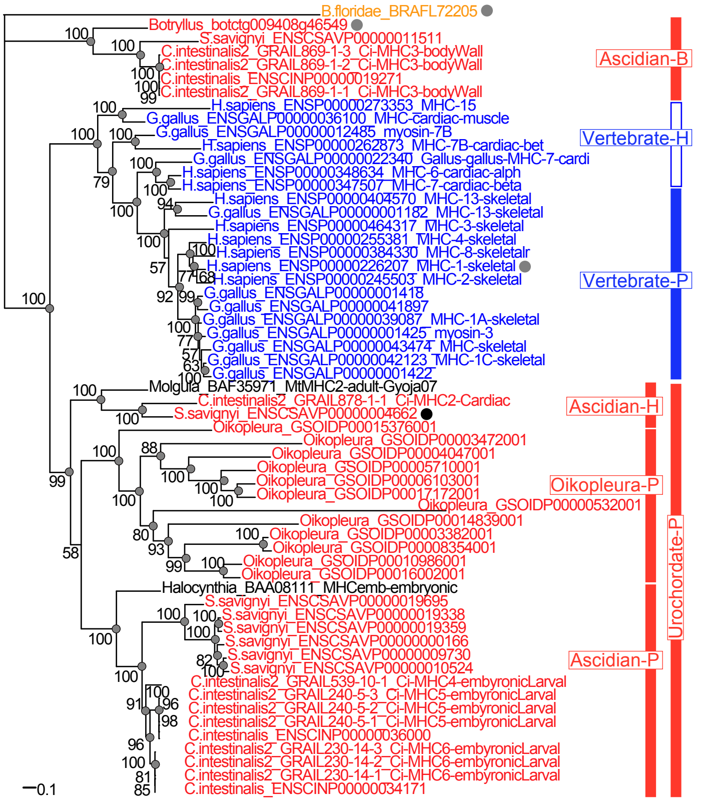

Fig. S2B

3. Rearranged gene tree

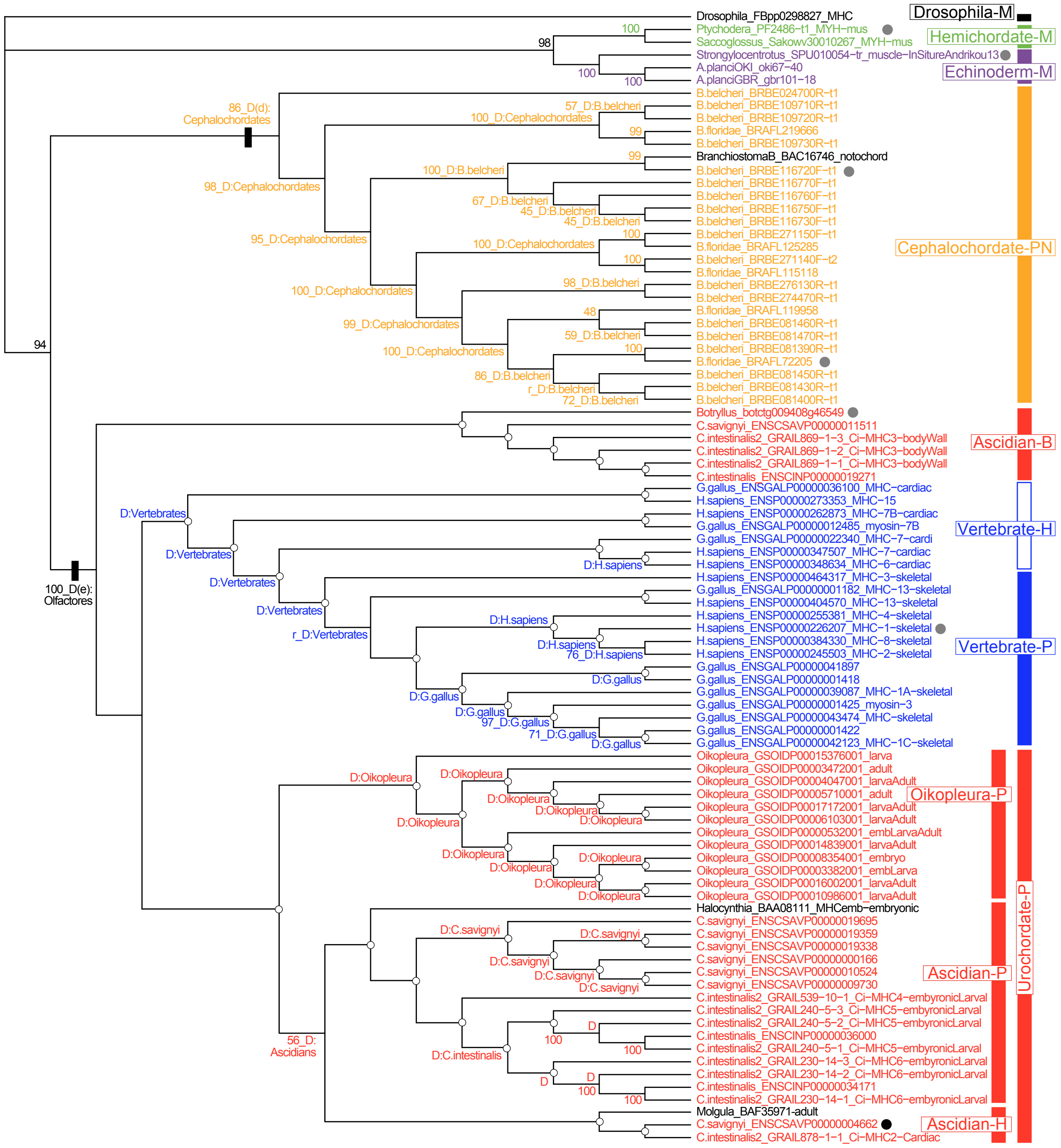

Fig. S2B
(continued)

(C) Tropomyosin

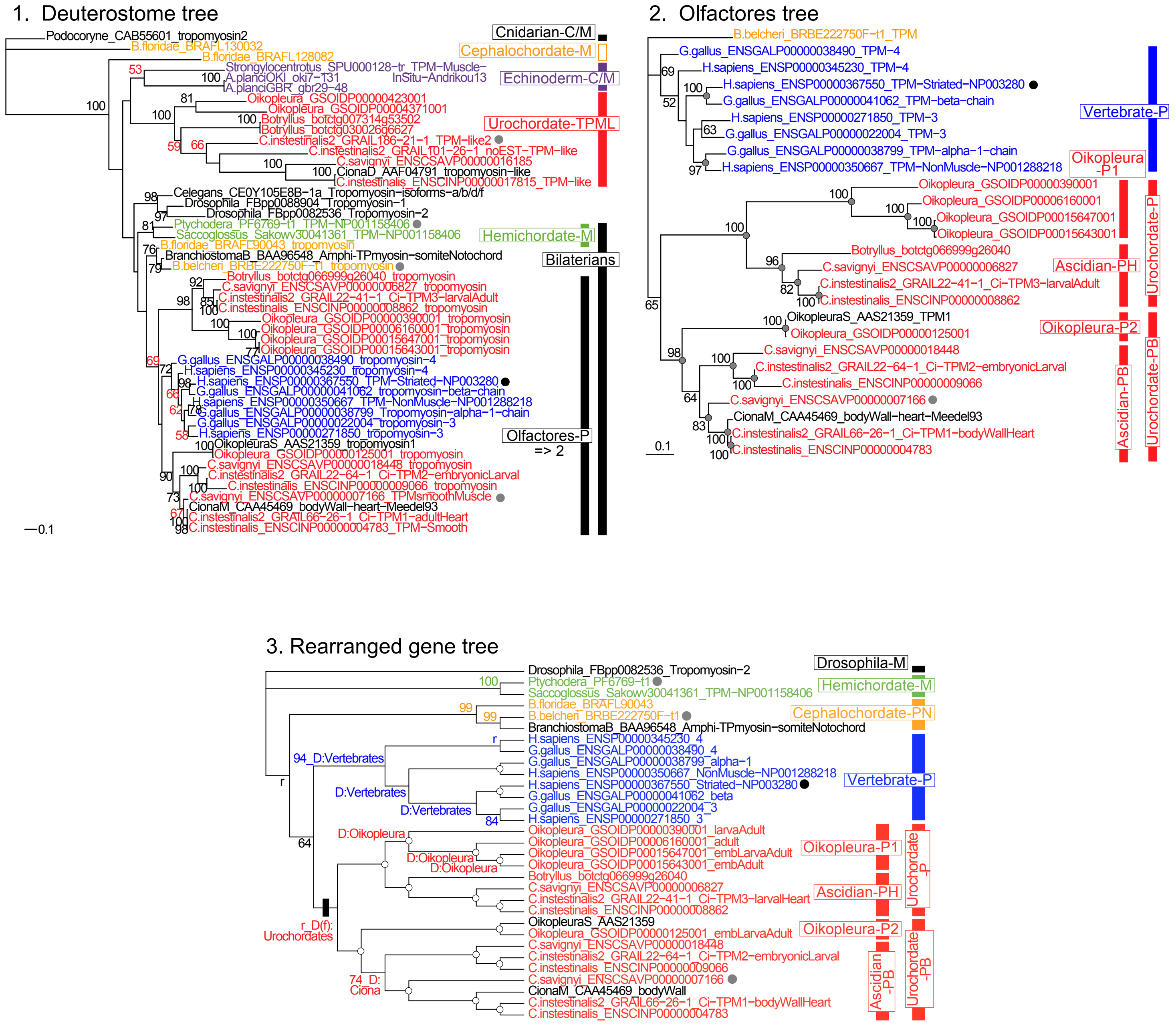

Fig. S2C

4. Amino acid sequence alignment of tropomyosin genes

(a) N terminal region

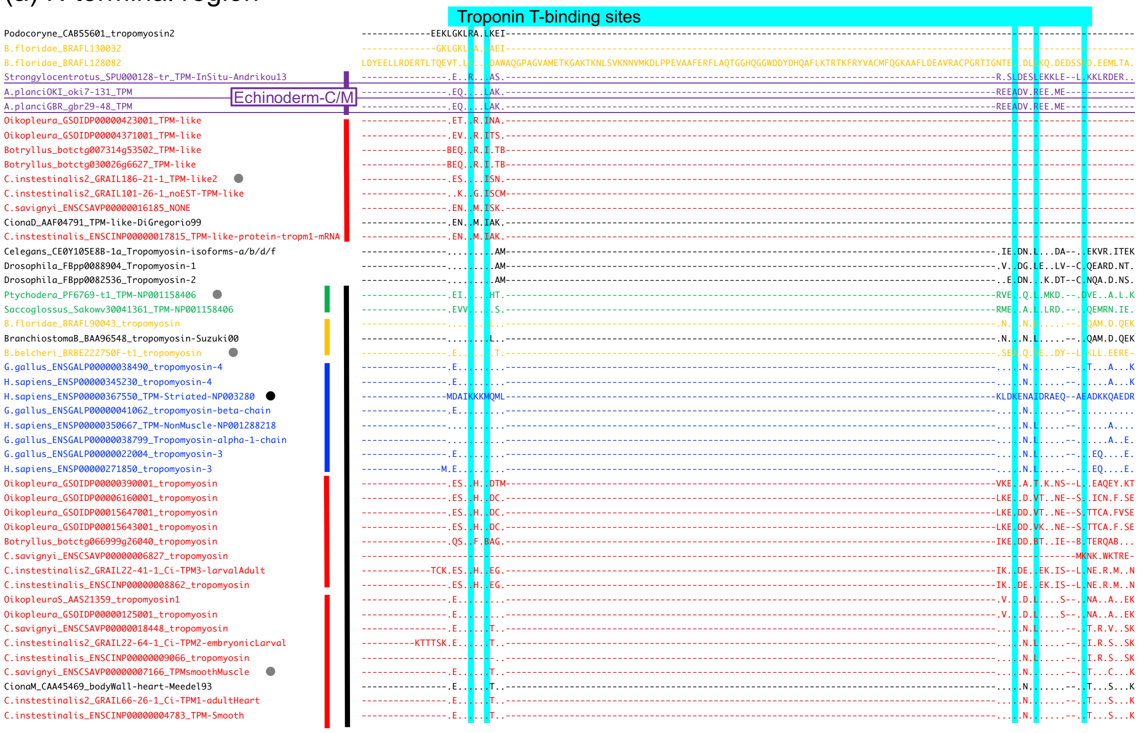

(b) C terminal region

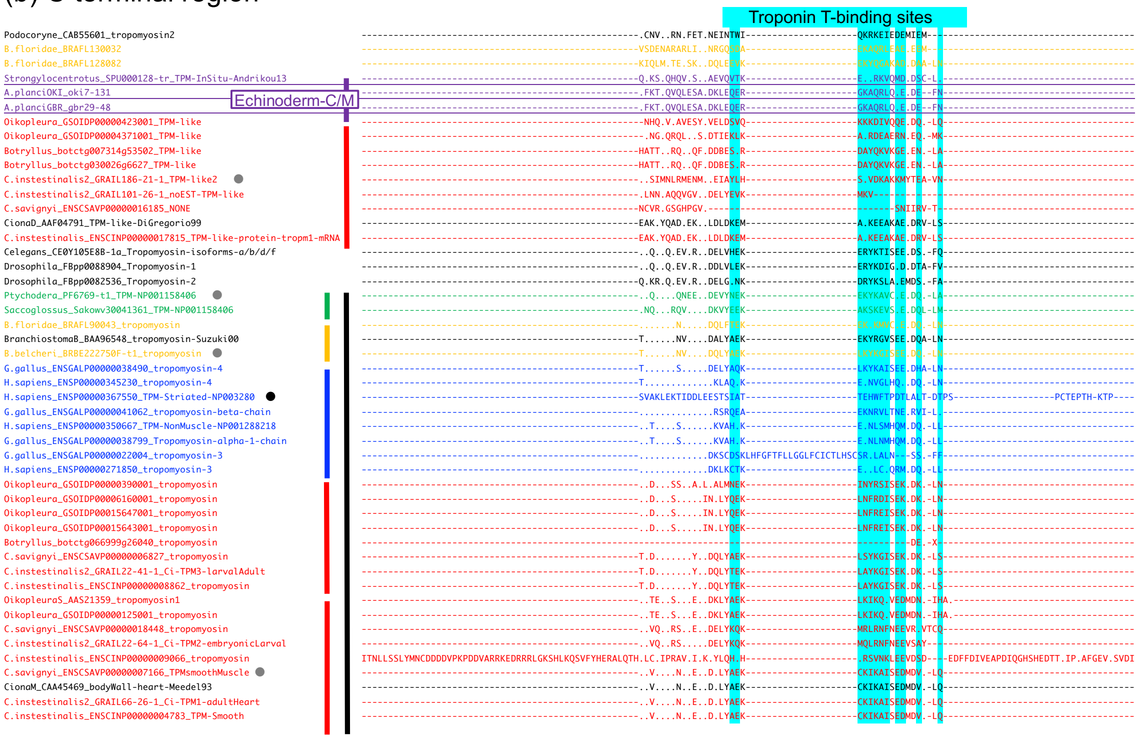

Fig. S2C
(continued)

(D) Troponin I

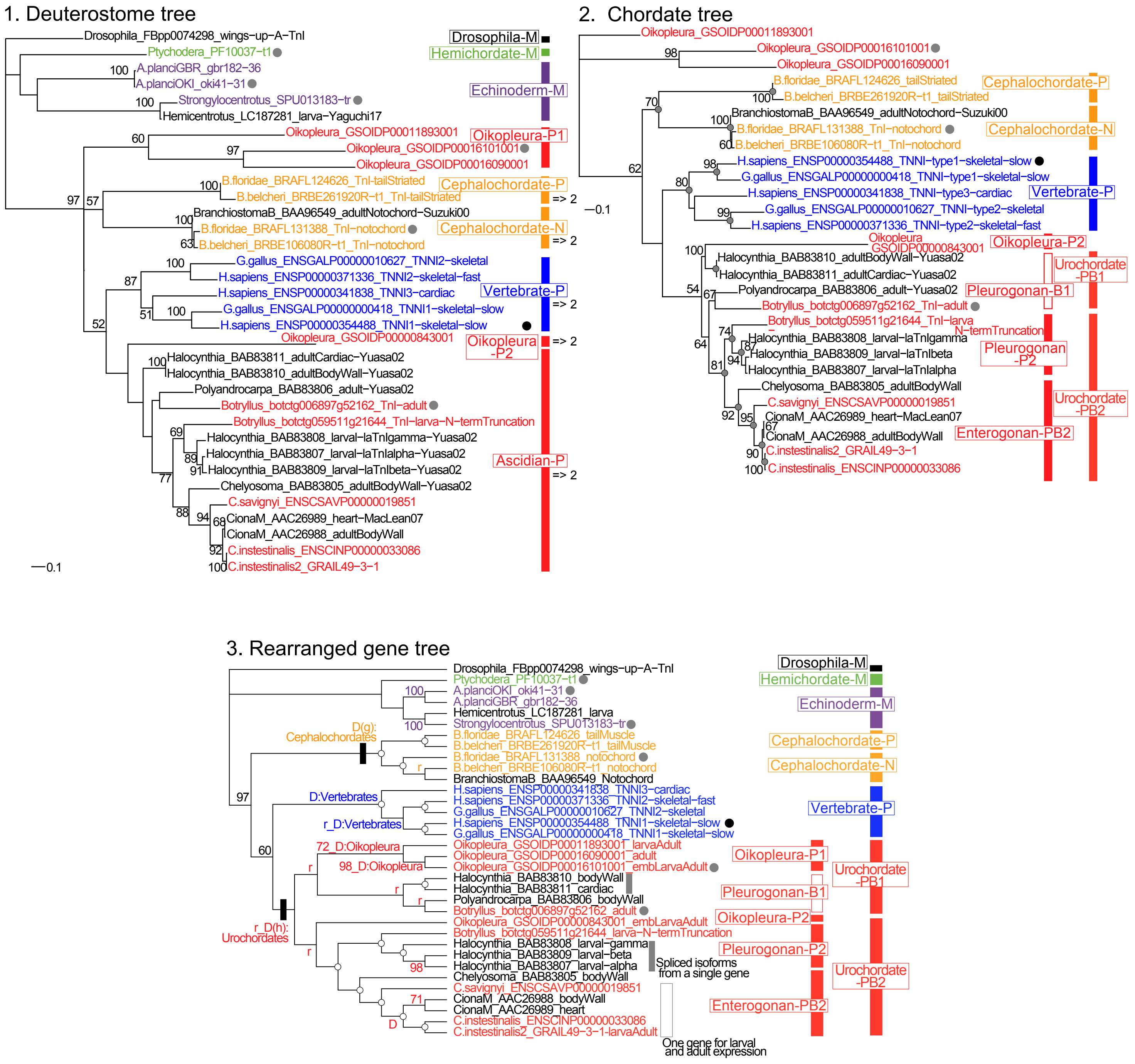

Fig. S2D

4. Amino acid sequence alignment of troponin I genes
(a) N terminal region

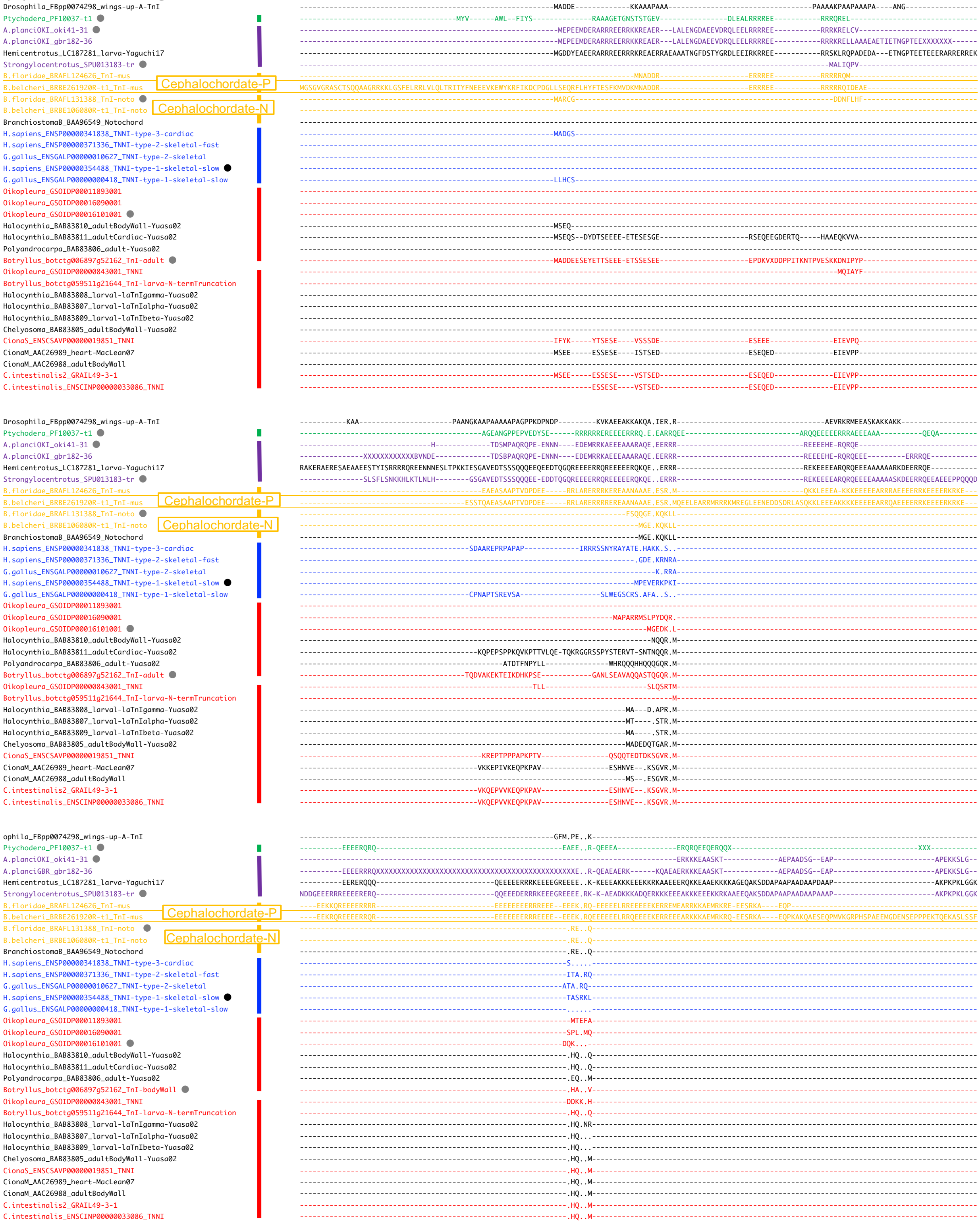

Fig. S2D
(continued)

(b) Troponin T-binding region

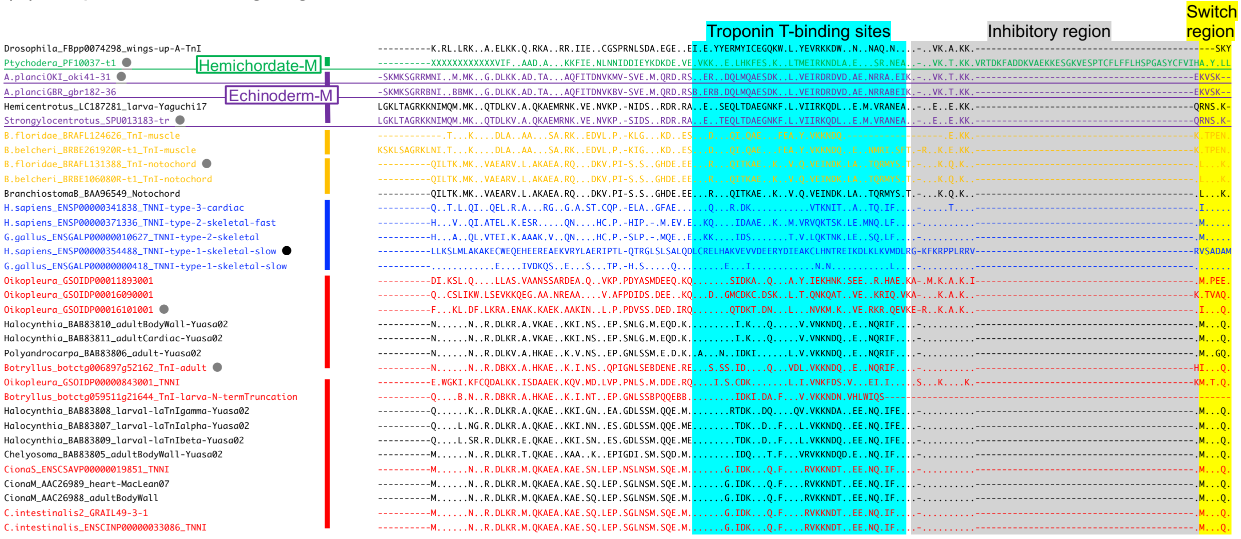

(c) C terminal region

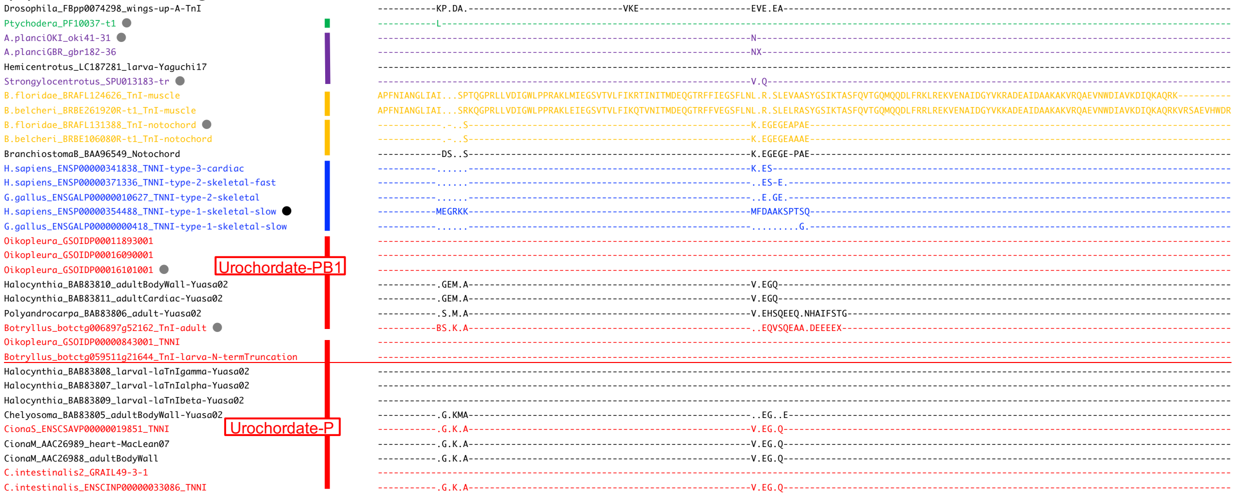

Fig. S2D
(continued)

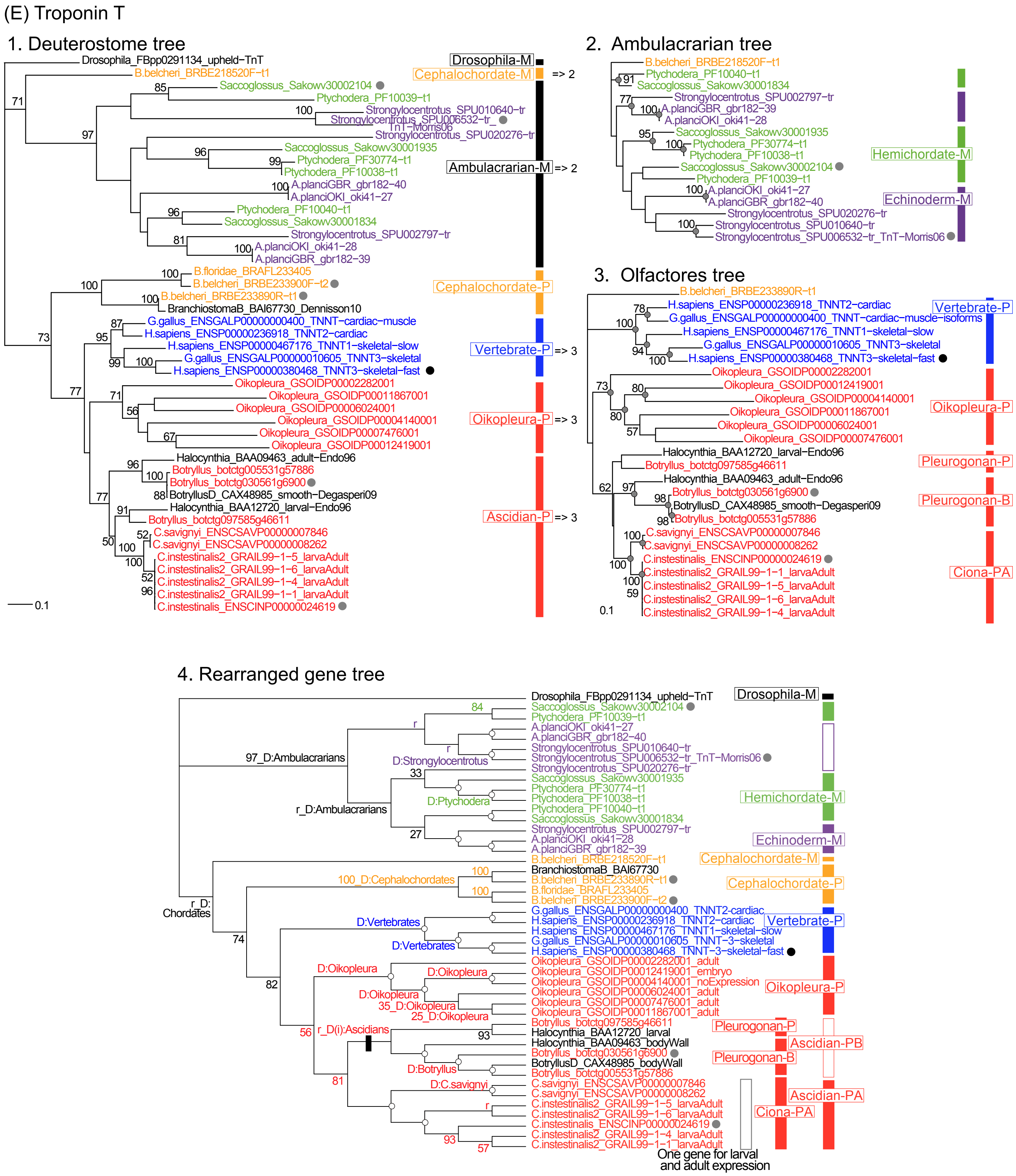

(E) Troponin T
1. Deuterostome tree
Drosophila_FBpp0291134_upheld−TnT
B.belcheri_BRBE218520F−t1
Saccoglossus_Sakowv30002104
Ptychodera_PF10039−t1
Strongylocentrotus_SPU010640−tr
Strongylocentrotus_SPU006532−tr_TnT−Morris06
Strongylocentrotus_SPU020276−tr
Saccoglossus_Sakowv30001935
Ptychodera_PF30774−t1
Ptychodera_PF10038−t1
A.planciGBR_gbr182−40
A.planciOKI_oki41−27
Ptychodera_PF10040−t1
Saccoglossus_Sakowv30001834
Strongylocentrotus_SPU002797−tr
A.planciOKI_oki41−28
A.planciGBR_gbr182−39
B.floridae_BRAFL233405
B.belcheri_BRBE233900F−t2
B.belcheri_BRBE233890R−t1
BranchiostomaB_BAI67730_Dennisson10
G.gallus_ENSGALP00000000400_TNNT−cardiac−muscle
H.sapiens_ENSP00000236918_TNNT2−cardiac
H.sapiens_ENSP00000467176_TNNT1−skeletal−slow
G.gallus_ENSGALP00000010605_TNNT3−skeletal
H.sapiens_ENSP00000380468_TNNT3−skeletal−fast
Oikopleura_GSOIDP00002282001
Oikopleura_GSOIDP00011867001
Oikopleura_GSOIDP00006024001
Oikopleura_GSOIDP00004140001
Oikopleura_GSOIDP00007476001
Oikopleura_GSOIDP00012419001
Halocynthia_BAA09463_adult−Endo96
Botryllus_botctg005531g57886
Botryllus_botctg030561g6900
BotryllusD_CAX48985_smooth−Degasperi09
Halocynthia_BAA12720_larval−Endo96
Botryllus_botctg097585g46611
C.savignyi_ENSCSAVP00000007846
C.savignyi_ENSCSAVP00000008262
C.instestinalis2_GRAIL99−1−5_larvaAdult
C.instestinalis2_GRAIL99−1−6_larvaAdult
C.instestinalis2_GRAIL99−1−4_larvaAdult
C.instestinalis2_GRAIL99−1−1_larvaAdult
C.instestinalis_ENSCINP00000024619
0.1
Drosophila-M
Cephalochordate-M => 2
Ambulacrarian-M => 2
Cephalochordate-P
Vertebrate-P => 3
Oikopleura-P => 3
Ascidian-P => 3
2. Ambulacrarian tree
B.belcheri_BRBE218520F−t1
Ptychodera_PF10040−t1
Saccoglossus_Sakowv30001834
Strongylocentrotus_SPU002797−tr
A.planciGBR_gbr182−39
A.planciOKI_oki41−28
Saccoglossus_Sakowv30001935
Ptychodera_PF30774−t1
Ptychodera_PF10038−t1
Saccoglossus_Sakowv30002104
Ptychodera_PF10039−t1
A.planciOKI_oki41−27
A.planciGBR_gbr182−40
Strongylocentrotus_SPU020276−tr
Strongylocentrotus_SPU010640−tr
Strongylocentrotus_SPU006532−tr_TnT−Morris06
Hemichordate-M
Echinoderm-M
3. Olfactores tree
B.belcheri_BRBE233890R−t1
H.sapiens_ENSP00000236918_TNNT2−cardiac
G.gallus_ENSGALP00000000400_TNNT−cardiac−muscle−isoforms
H.sapiens_ENSP00000467176_TNNT1−skeletal−slow
G.gallus_ENSGALP00000010605_TNNT3−skeletal
H.sapiens_ENSP00000380468_TNNT3−skeletal−fast
Oikopleura_GSOIDP00002282001
Oikopleura_GSOIDP00012419001
Oikopleura_GSOIDP00004140001
Oikopleura_GSOIDP00011867001
Oikopleura_GSOIDP00006024001
Oikopleura_GSOIDP00007476001
Halocynthia_BAA12720_larval−Endo96
Botryllus_botctg097585g46611
Halocynthia_BAA09463_adult−Endo96
Botryllus_botctg030561g6900
BotryllusD_CAX48985_smooth−Degasperi09
Botryllus_botctg005531g57886
C.savignyi_ENSCSAVP00000007846
C.savignyi_ENSCSAVP00000008262
C.instestinalis_ENSCINP00000024619
C.instestinalis2_GRAIL99−1−1_larvaAdult
C.instestinalis2_GRAIL99−1−5_larvaAdult
C.instestinalis2_GRAIL99−1−6_larvaAdult
C.instestinalis2_GRAIL99−1−4_larvaAdult
0.1
Vertebrate-P
Oikopleura-P
Pleurogonan-P
Pleurogonan-B
Ciona-PA
4. Rearranged gene tree
Drosophila_FBpp0291134_upheld−TnT
Saccoglossus_Sakowv30002104
Ptychodera_PF10039−t1
A.planciOKI_oki41−27
A.planciGBR_gbr182−40
Strongylocentrotus_SPU010640−tr
Strongylocentrotus_SPU006532−tr_TnT−Morris06
Strongylocentrotus_SPU020276−tr
Saccoglossus_Sakowv30001935
Ptychodera_PF30774−t1
Ptychodera_PF10038−t1
Ptychodera_PF10040−t1
Saccoglossus_Sakowv30001834
Strongylocentrotus_SPU002797−tr
A.planciOKI_oki41−28
A.planciGBR_gbr182−39
B.belcheri_BRBE218520F−t1
BranchiostomaB_BAI67730
B.belcheri_BRBE233890R−t1
B.floridae_BRAFL233405
B.belcheri_BRBE233900F−t2
G.gallus_ENSGALP00000000400_TNNT2-cardiac
H.sapiens_ENSP00000236918_TNNT2-cardiac
H.sapiens_ENSP00000467176_TNNT1−skeletal−slow
G.gallus_ENSGALP00000010605_TNNT−3−skeletal
H.sapiens_ENSP00000380468_TNNT−3−skeletal−fast
Oikopleura_GSOIDP00002282001_adult
Oikopleura_GSOIDP00012419001_embryo
Oikopleura_GSOIDP00004140001_noExpression
Oikopleura_GSOIDP00006024001_adult
Oikopleura_GSOIDP00007476001_adult
Oikopleura_GSOIDP00011867001_adult
Botryllus_botctg097585g46611
Halocynthia_BAA12720_larval
Halocynthia_BAA09463_bodyWall
Botryllus_botctg030561g6900
BotryllusD_CAX48985_bodyWall
Botryllus_botctg005531g57886
C.savignyi_ENSCSAVP00000007846
C.savignyi_ENSCSAVP00000008262
C.instestinalis2_GRAIL99−1−5_larvaAdult
C.instestinalis2_GRAIL99−1−6_larvaAdult
C.instestinalis_ENSCINP00000024619
C.instestinalis2_GRAIL99−1−4_larvaAdult
C.instestinalis2_GRAIL99−1−1_larvaAdult
97_D:Ambulacrarians
r_D:Ambulacrarians
D:Strongylocentrotus
D:Ptychodera
r_D: Chordates
100_D:Cephalochordates
D:Vertebrates
D:Oikopleura
35_D:Oikopleura
25_D:Oikopleura
r_D(i):Ascidians
D:Botryllus
D:C.savignyi
Drosophila-M
Hemichordate-M
Echinoderm-M
Cephalochordate-M
Cephalochordate-P
Vertebrate-P
Oikopleura-P
Pleurogonan-P
Ascidian-PB
Pleurogonan-B
Ascidian-PA
Ciona-PA
One gene for larval and adult expression

Fig. S2E

5. Amino acid sequence alignment of troponin T genes

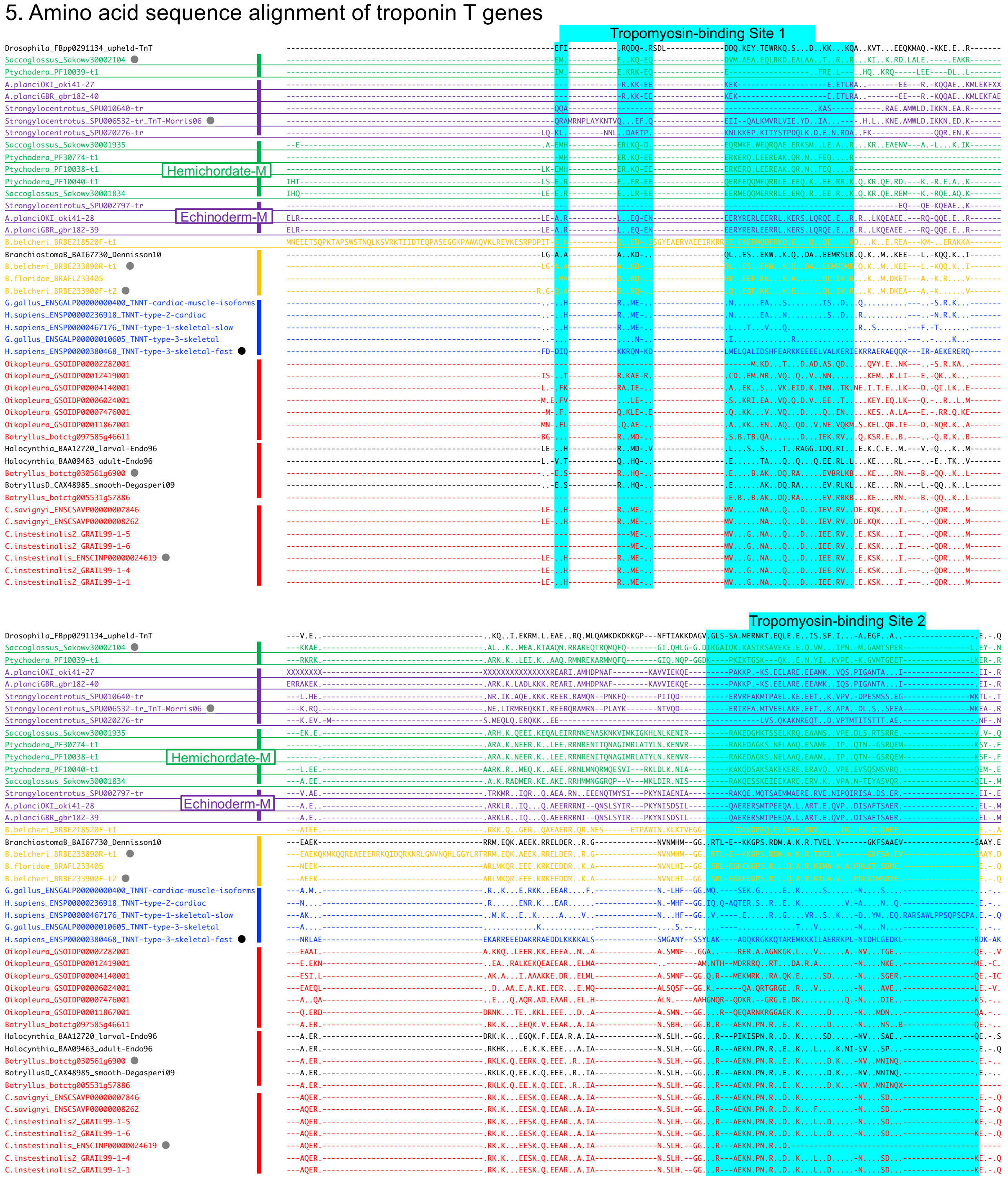

Fig. S2E
(continued)

(F) Troponin C

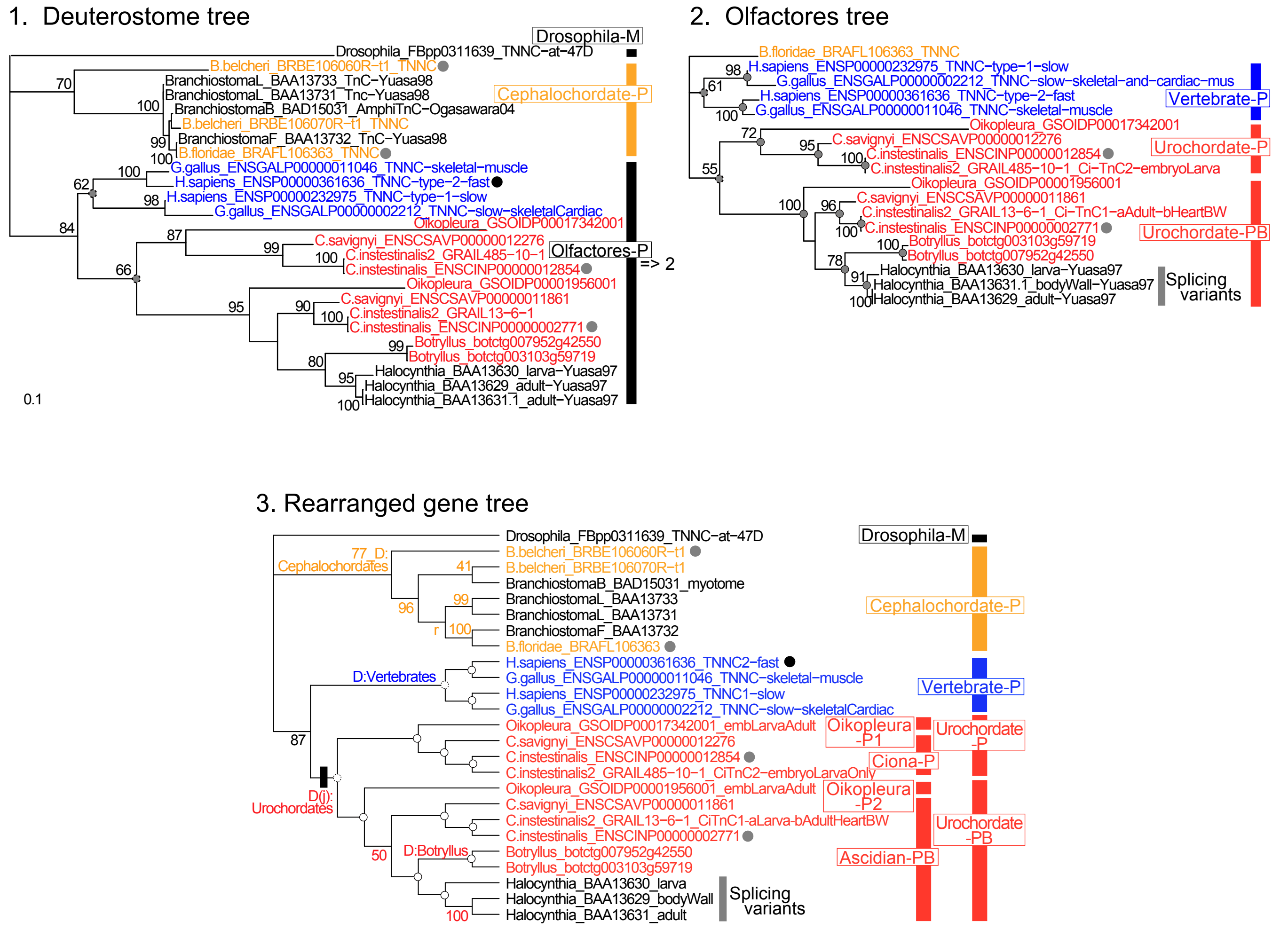

Fig. S2F

(G) Tropomodulin

1. Deuterostome tree

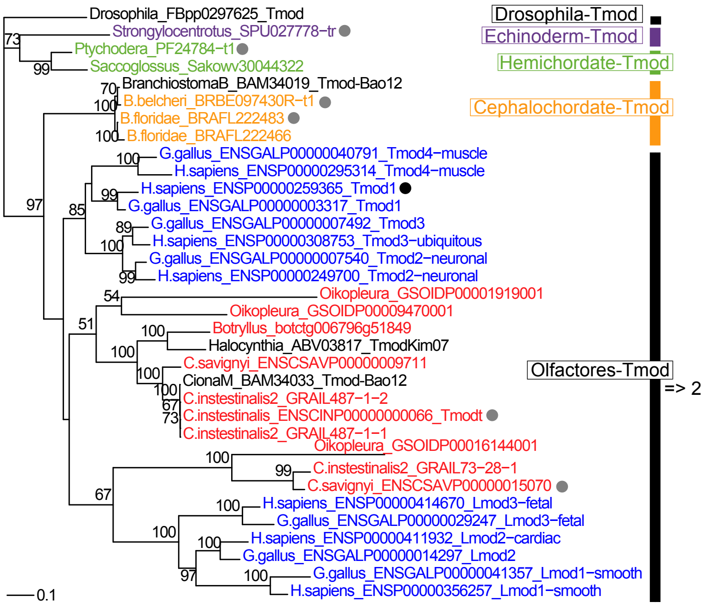

2. Olfactores tree

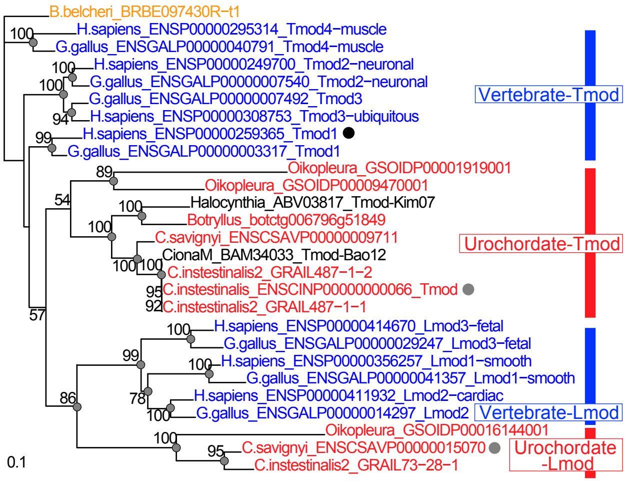

3. Rearranged gene tree

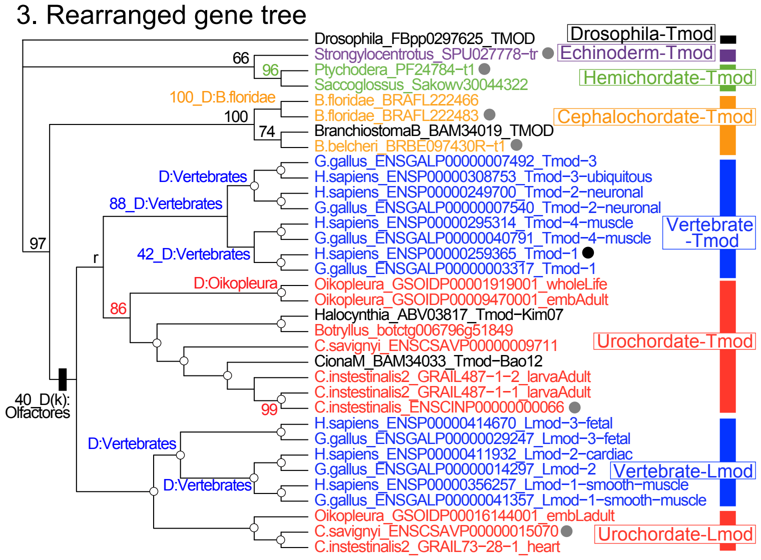

Fig. S2G

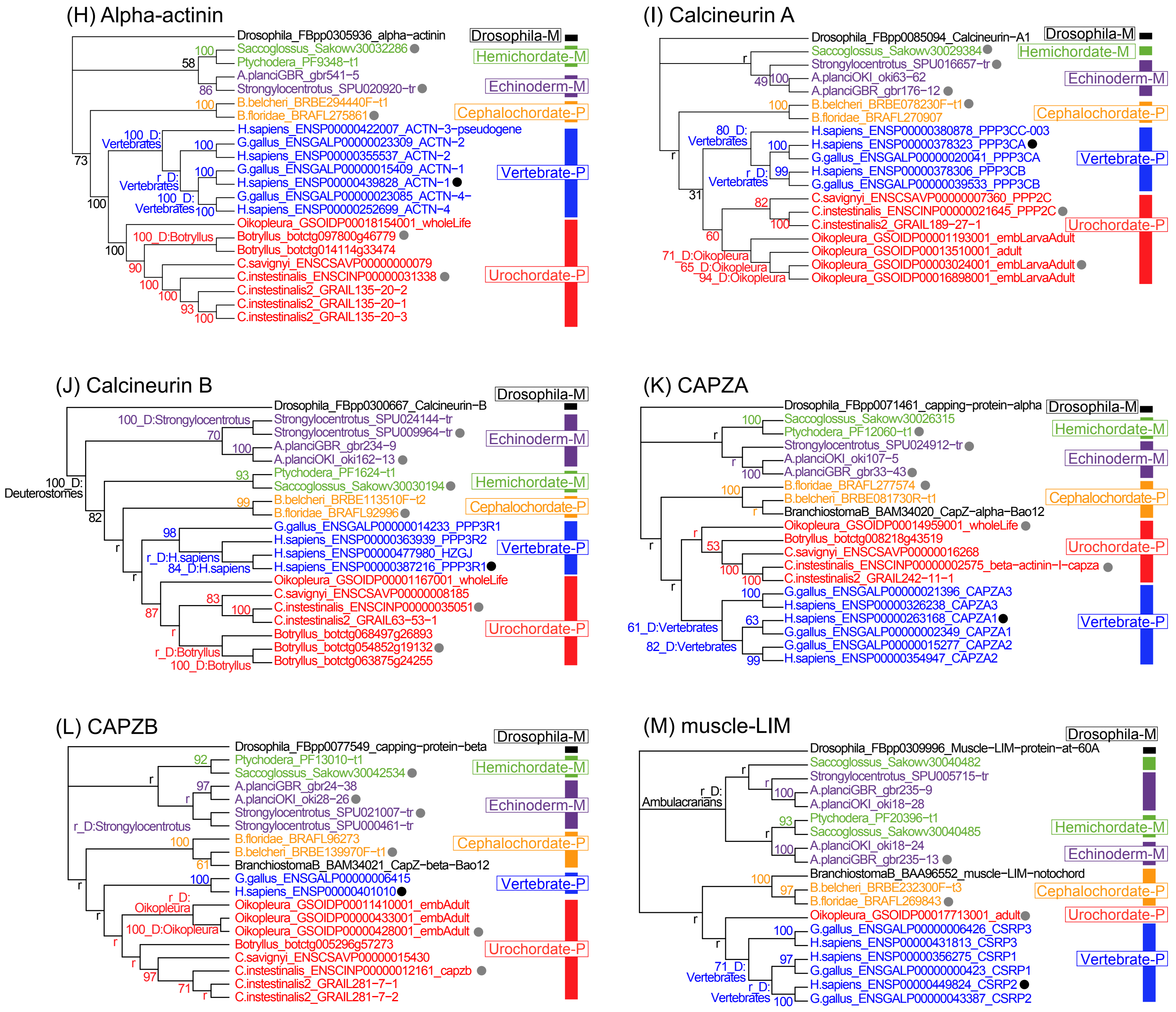
(H) Alpha-actinin
Drosophila_FBpp0305936_alpha-actinin
Saccoglossus_Sakowv30032286
Ptychodera_PF9348-t1
A.planciGBR_gbr541-5
Strongylocentrotus_SPU020920-tr
B.belcheri_BRBE294440F-t1
B.floridae_BRAFL275861
H.sapiens_ENSP00000422007_ACTN-3-pseudogene
G.gallus_ENSGALP00000023309_ACTN-2
H.sapiens_ENSP00000355537_ACTN-2
G.gallus_ENSGALP00000015409_ACTN-1
H.sapiens_ENSP00000439828_ACTN-1
G.gallus_ENSGALP00000023085_ACTN-4-
H.sapiens_ENSP00000252699_ACTN-4
Oikopleura_GSOIDP00018154001_wholeLife
Botryllus_botctg097800g46779
Botryllus_botctg014114g33474
C.savignyi_ENSCSAVP00000000079
C.instestinalis_ENSCINP00000031338
C.instestinalis2_GRAIL135-20-2
C.instestinalis2_GRAIL135-20-1
C.instestinalis2_GRAIL135-20-3
100_D: Vertebrates
r_D: Vertebrates
100_D: Vertebrates
100_D:Botryllus
Drosophila-M
Hemichordate-M
Echinoderm-M
Cephalochordate-P
Vertebrate-P
Urochordate-P
(I) Calcineurin A
Drosophila_FBpp0085094_Calcineurin-A1
Saccoglossus_Sakowv30029384
Strongylocentrotus_SPU016657-tr
A.planciOKI_oki63-62
A.planciGBR_gbr176-12
B.belcheri_BRBE078230F-t1
B.floridae_BRAFL270907
H.sapiens_ENSP00000380878_PPP3CC-003
H.sapiens_ENSP00000378323_PPP3CA
G.gallus_ENSGALP00000020041_PPP3CA
H.sapiens_ENSP00000378306_PPP3CB
G.gallus_ENSGALP00000039533_PPP3CB
C.savignyi_ENSCSAVP00000007360_PPP2C
C.instestinalis_ENSCINP00000021645_PPP2C
C.instestinalis2_GRAIL189-27-1
Oikopleura_GSOIDP00001193001_embLarvaAdult
Oikopleura_GSOIDP00013510001_adult
Oikopleura_GSOIDP00003024001_embLarvaAdult
Oikopleura_GSOIDP00016898001_embLarvaAdult
80_D: Vertebrates
r_D: Vertebrates
71_D:Oikopleura
65_D:Oikopleura
94_D:Oikopleura
Drosophila-M
Hemichordate-M
Echinoderm-M
Cephalochordate-P
Vertebrate-P
Urochordate-P
(J) Calcineurin B
Drosophila_FBpp0300667_Calcineurin-B
Strongylocentrotus_SPU024144-tr
Strongylocentrotus_SPU009964-tr
A.planciGBR_gbr234-9
A.planciOKI_oki162-13
Ptychodera_PF1624-t1
Saccoglossus_Sakowv30030194
B.belcheri_BRBE113510F-t2
B.floridae_BRAFL92996
G.gallus_ENSGALP00000014233_PPP3R1
H.sapiens_ENSP00000363939_PPP3R2
H.sapiens_ENSP00000477980_HZGJ
H.sapiens_ENSP00000387216_PPP3R1
Oikopleura_GSOIDP00001167001_wholeLife
C.savignyi_ENSCSAVP00000008185
C.instestinalis_ENSCINP00000035051
C.instestinalis2_GRAIL63-53-1
Botryllus_botctg068497g26893
Botryllus_botctg054852g19132
Botryllus_botctg063875g24255
100_D:Strongylocentrotus
100_D: Deuterostomes
r_D:H.sapiens
84_D:H.sapiens
r_D:Botryllus
100_D:Botryllus
Drosophila-M
Echinoderm-M
Hemichordate-M
Cephalochordate-P
Vertebrate-P
Urochordate-P
(K) CAPZA
Drosophila_FBpp0071461_capping-protein-alpha
Saccoglossus_Sakowv30026315
Ptychodera_PF12060-t1
Strongylocentrotus_SPU024912-tr
A.planciOKI_oki107-5
A.planciGBR_gbr33-43
B.floridae_BRAFL277574
B.belcheri_BRBE081730R-t1
BranchiostomaB_BAM34020_CapZ-alpha-Bao12
Oikopleura_GSOIDP00014959001_wholeLife
Botryllus_botctg008218g43519
C.savignyi_ENSCSAVP00000016268
C.instestinalis_ENSCINP00000002575_beta-actinin-I-capza
C.instestinalis2_GRAIL242-11-1
G.gallus_ENSGALP00000021396_CAPZA3
H.sapiens_ENSP00000326238_CAPZA3
H.sapiens_ENSP00000263168_CAPZA1
G.gallus_ENSGALP00000002349_CAPZA1
G.gallus_ENSGALP00000015277_CAPZA2
H.sapiens_ENSP00000354947_CAPZA2
61_D:Vertebrates
82_D:Vertebrates
Drosophila-M
Hemichordate-M
Echinoderm-M
Cephalochordate-P
Urochordate-P
Vertebrate-P
(L) CAPZB
Drosophila_FBpp0077549_capping-protein-beta
Ptychodera_PF13010-t1
Saccoglossus_Sakowv30042534
A.planciGBR_gbr24-38
A.planciOKI_oki28-26
Strongylocentrotus_SPU021007-tr
Strongylocentrotus_SPU000461-tr
B.floridae_BRAFL96273
B.belcheri_BRBE139970F-t1
BranchiostomaB_BAM34021_CapZ-beta-Bao12
G.gallus_ENSGALP00000006415
H.sapiens_ENSP00000401010
Oikopleura_GSOIDP00011410001_embAdult
Oikopleura_GSOIDP00000433001_embAdult
Oikopleura_GSOIDP00000428001_embAdult
Botryllus_botctg005296g57273
C.savignyi_ENSCSAVP00000015430
C.instestinalis_ENSCINP00000012161_capzb
C.instestinalis2_GRAIL281-7-1
C.instestinalis2_GRAIL281-7-2
r_D:Strongylocentrotus
r_D: Oikopleura
100_D:Oikopleura
Drosophila-M
Hemichordate-M
Echinoderm-M
Cephalochordate-P
Vertebrate-P
Urochordate-P
(M) muscle-LIM
Drosophila_FBpp0309996_Muscle-LIM-protein-at-60A
Saccoglossus_Sakowv30040482
Strongylocentrotus_SPU005715-tr
A.planciGBR_gbr235-9
A.planciOKI_oki18-28
Ptychodera_PF20396-t1
Saccoglossus_Sakowv30040485
A.planciOKI_oki18-24
A.planciGBR_gbr235-13
BranchiostomaB_BAA96552_muscle-LIM-notochord
B.belcheri_BRBE232300F-t3
B.floridae_BRAFL269843
Oikopleura_GSOIDP00017713001_adult
G.gallus_ENSGALP00000006426_CSRP3
H.sapiens_ENSP00000431813_CSRP3
H.sapiens_ENSP00000356275_CSRP1
G.gallus_ENSGALP00000000423_CSRP1
H.sapiens_ENSP00000449824_CSRP2
G.gallus_ENSGALP00000043387_CSRP2
r_D: Ambulacrarians
71_D: Vertebrates
r_D: Vertebrates
Drosophila-M
Hemichordate-M
Echinoderm-M
Cephalochordate-P
Urochordate-P
Vertebrate-P

Fig. S2H-M

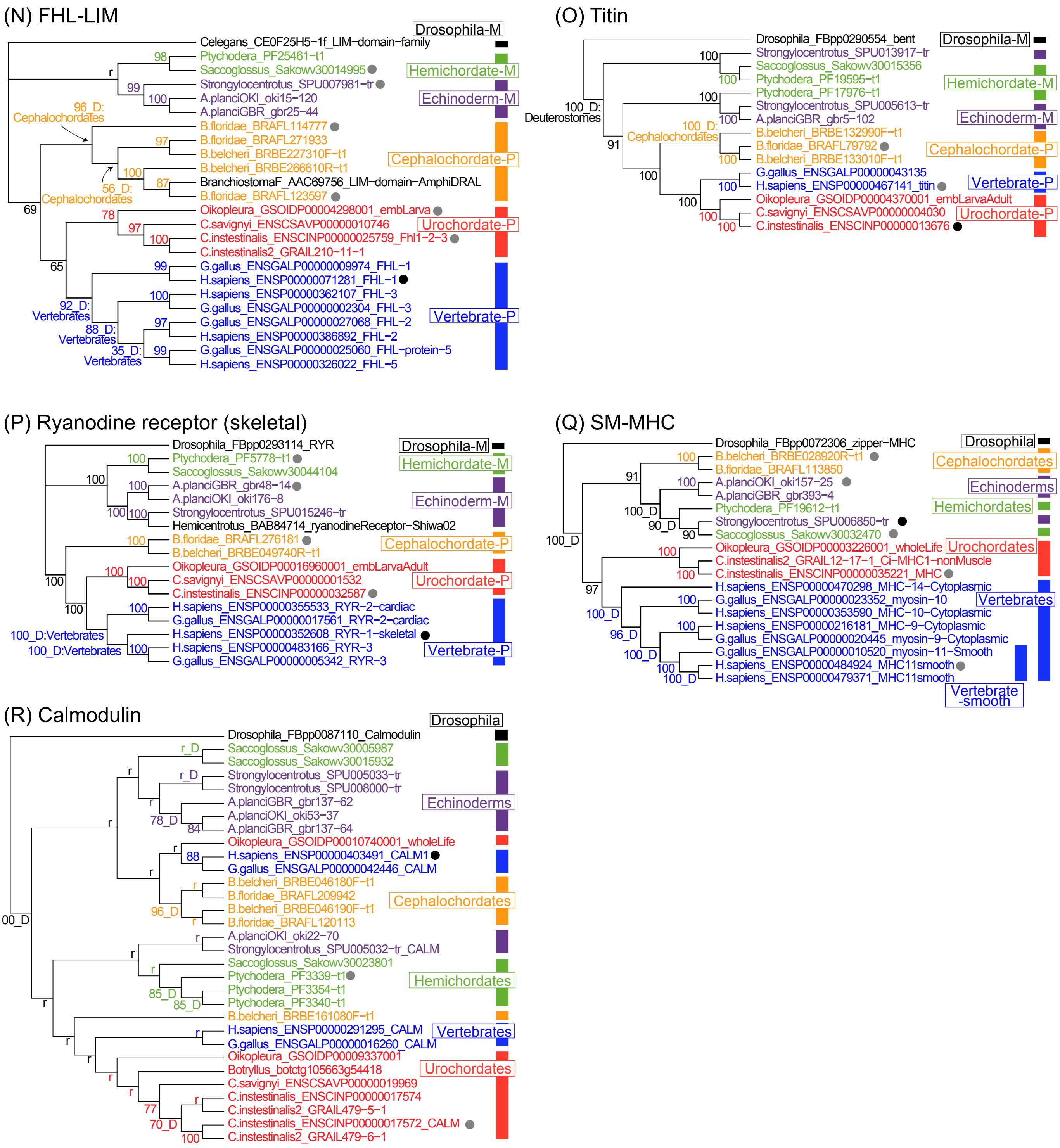
(N) FHL-LIM
Drosophila-M
Celegans_CE0F25H5−1f_LIM−domain−family
Ptychodera_PF25461−t1
Saccoglossus_Sakowv30014995
Hemichordate-M
Strongylocentrotus_SPU007981−tr
A.planciOKI_oki15−120
Echinoderm-M
A.planciGBR_gbr25−44
96_D:
Cephalochordates
B.floridae_BRAFL114777
B.floridae_BRAFL271933
B.belcheri_BRBE227310F−t1
Cephalochordate-P
B.belcheri_BRBE266610R−t1
BranchiostomaF_AAC69756_LIM−domain−AmphiDRAL
56_D:
Cephalochordates
B.floridae_BRAFL123597
Oikopleura_GSOIDP00004298001_embLarva
C.savignyi_ENSCSAVP00000010746
Urochordate-P
C.instestinalis_ENSCINP00000025759_Fhl1−2−3
C.instestinalis2_GRAIL210−11−1
G.gallus_ENSGALP00000009974_FHL−1
H.sapiens_ENSP00000071281_FHL−1
H.sapiens_ENSP00000362107_FHL−3
92_D:
Vertebrates
G.gallus_ENSGALP00000002304_FHL−3
Vertebrate-P
88_D:
Vertebrates
G.gallus_ENSGALP00000027068_FHL−2
H.sapiens_ENSP00000386892_FHL−2
35_D:
Vertebrates
G.gallus_ENSGALP00000025060_FHL−protein−5
H.sapiens_ENSP00000326022_FHL−5
(O) Titin
Drosophila_FBpp0290554_bent
Drosophila-M
Strongylocentrotus_SPU013917−tr
Saccoglossus_Sakowv30015356
Ptychodera_PF19595−t1
Hemichordate-M
Ptychodera_PF17976−t1
100_D:
Deuterostomes
Strongylocentrotus_SPU005613−tr
A.planciGBR_gbr5−102
Echinoderm-M
100_D:
Cephalochordates
B.belcheri_BRBE132990F−t1
B.floridae_BRAFL79792
Cephalochordate-P
B.belcheri_BRBE133010F−t1
G.gallus_ENSGALP00000043135
H.sapiens_ENSP00000467141_titin
Vertebrate-P
Oikopleura_GSOIDP00004370001_embLarvaAdult
C.savignyi_ENSCSAVP00000004030
Urochordate-P
C.instestinalis_ENSCINP00000013676
(P) Ryanodine receptor (skeletal)
Drosophila_FBpp0293114_RYR
Drosophila-M
Ptychodera_PF5778−t1
Hemichordate-M
Saccoglossus_Sakowv30044104
A.planciGBR_gbr48−14
A.planciOKI_oki176−8
Echinoderm-M
Strongylocentrotus_SPU015246−tr
Hemicentrotus_BAB84714_ryanodineReceptor−Shiwa02
B.floridae_BRAFL276181
Cephalochordate-P
B.belcheri_BRBE049740R−t1
Oikopleura_GSOIDP00016960001_embLarvaAdult
C.savignyi_ENSCSAVP00000001532
Urochordate-P
C.instestinalis_ENSCINP00000032587
H.sapiens_ENSP00000355533_RYR−2−cardiac
G.gallus_ENSGALP00000017561_RYR−2−cardiac
100_D:Vertebrates
H.sapiens_ENSP00000352608_RYR−1−skeletal
100_D:Vertebrates
H.sapiens_ENSP00000483166_RYR−3
Vertebrate-P
G.gallus_ENSGALP00000005342_RYR−3
(Q) SM-MHC
Drosophila_FBpp0072306_zipper−MHC
Drosophila
B.belcheri_BRBE028920R−t1
Cephalochordates
B.floridae_BRAFL113850
A.planciOKI_oki157−25
Echinoderms
A.planciGBR_gbr393−4
Ptychodera_PF19612−t1
Hemichordates
Strongylocentrotus_SPU006850−tr
Saccoglossus_Sakowv30032470
Oikopleura_GSOIDP00003226001_wholeLife
Urochordates
C.instestinalis2_GRAIL12−17−1_Ci−MHC1−nonMuscle
C.instestinalis_ENSCINP00000035221_MHC
H.sapiens_ENSP00000470298_MHC−14−Cytoplasmic
G.gallus_ENSGALP00000023352_myosin−10
Vertebrates
H.sapiens_ENSP00000353590_MHC−10−Cytoplasmic
H.sapiens_ENSP00000216181_MHC−9−Cytoplasmic
G.gallus_ENSGALP00000020445_myosin−9−Cytoplasmic
G.gallus_ENSGALP00000010520_myosin−11−Smooth
H.sapiens_ENSP00000484924_MHC11smooth
H.sapiens_ENSP00000479371_MHC11smooth
Vertebrate
-smooth
(R) Calmodulin
Drosophila
Drosophila_FBpp0087110_Calmodulin
Saccoglossus_Sakowv30005987
Saccoglossus_Sakowv30015932
Strongylocentrotus_SPU005033−tr
Strongylocentrotus_SPU008000−tr
A.planciGBR_gbr137−62
Echinoderms
A.planciOKI_oki53−37
A.planciGBR_gbr137−64
Oikopleura_GSOIDP00010740001_wholeLife
H.sapiens_ENSP00000403491_CALM1
G.gallus_ENSGALP00000042446_CALM
B.belcheri_BRBE046180F−t1
B.floridae_BRAFL209942
Cephalochordates
B.belcheri_BRBE046190F−t1
B.floridae_BRAFL120113
A.planciOKI_oki22−70
Strongylocentrotus_SPU005032−tr_CALM
Saccoglossus_Sakowv30023801
Ptychodera_PF3339−t1
Hemichordates
Ptychodera_PF3354−t1
Ptychodera_PF3340−t1
B.belcheri_BRBE161080F−t1
H.sapiens_ENSP00000291295_CALM
Vertebrates
G.gallus_ENSGALP00000016260_CALM
Oikopleura_GSOIDP00009337001
Botryllus_botctg105663g54418
Urochordates
C.savignyi_ENSCSAVP00000019969
C.instestinalis_ENSCINP00000017574
C.instestinalis2_GRAIL479−5−1
C.instestinalis_ENSCINP00000017572_CALM
C.instestinalis2_GRAIL479−6−1

Fig. S2N-R

(S) MyoD

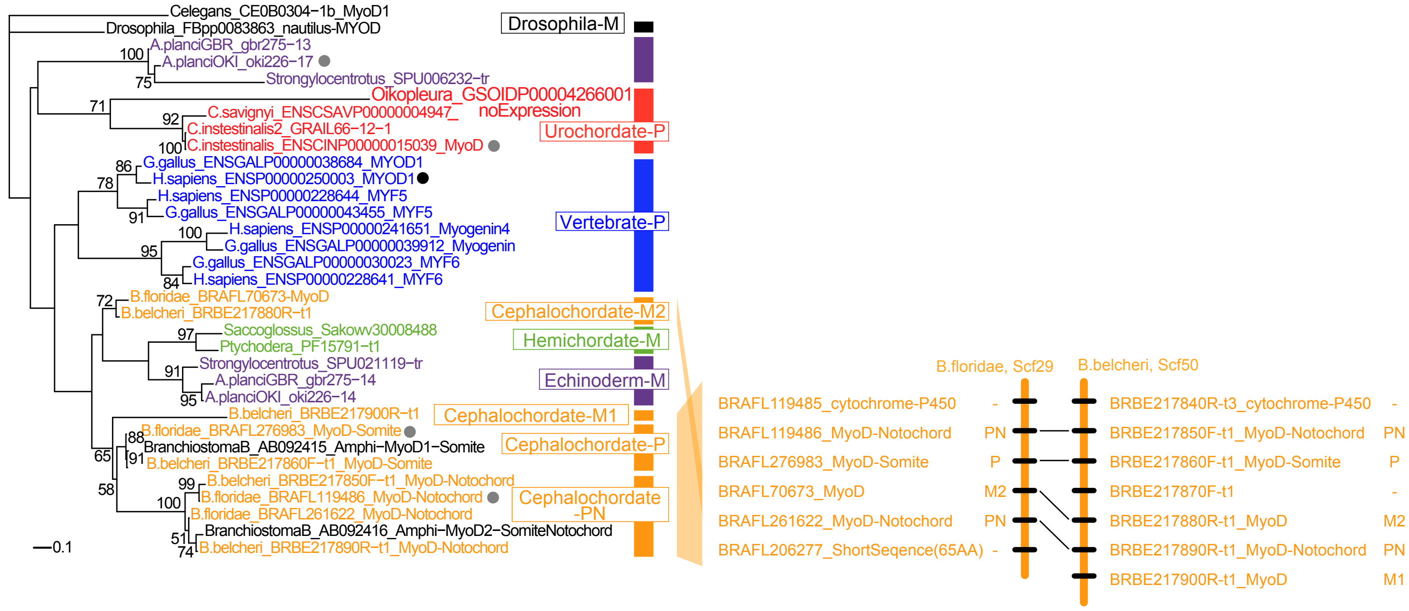

(T) SRF

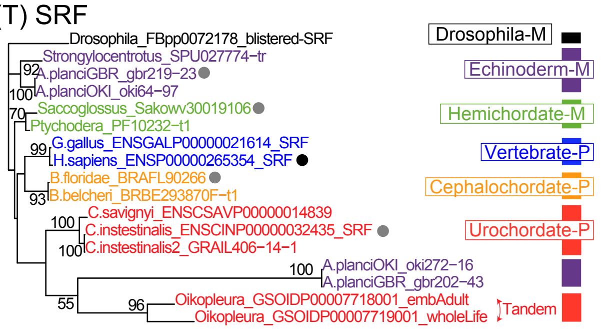

(U) MEF2

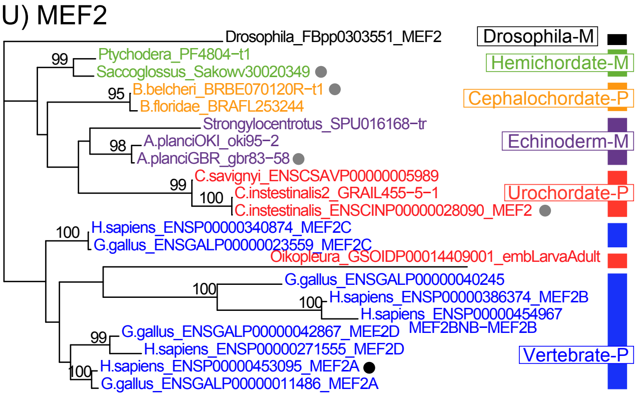

Fig. S2S-U

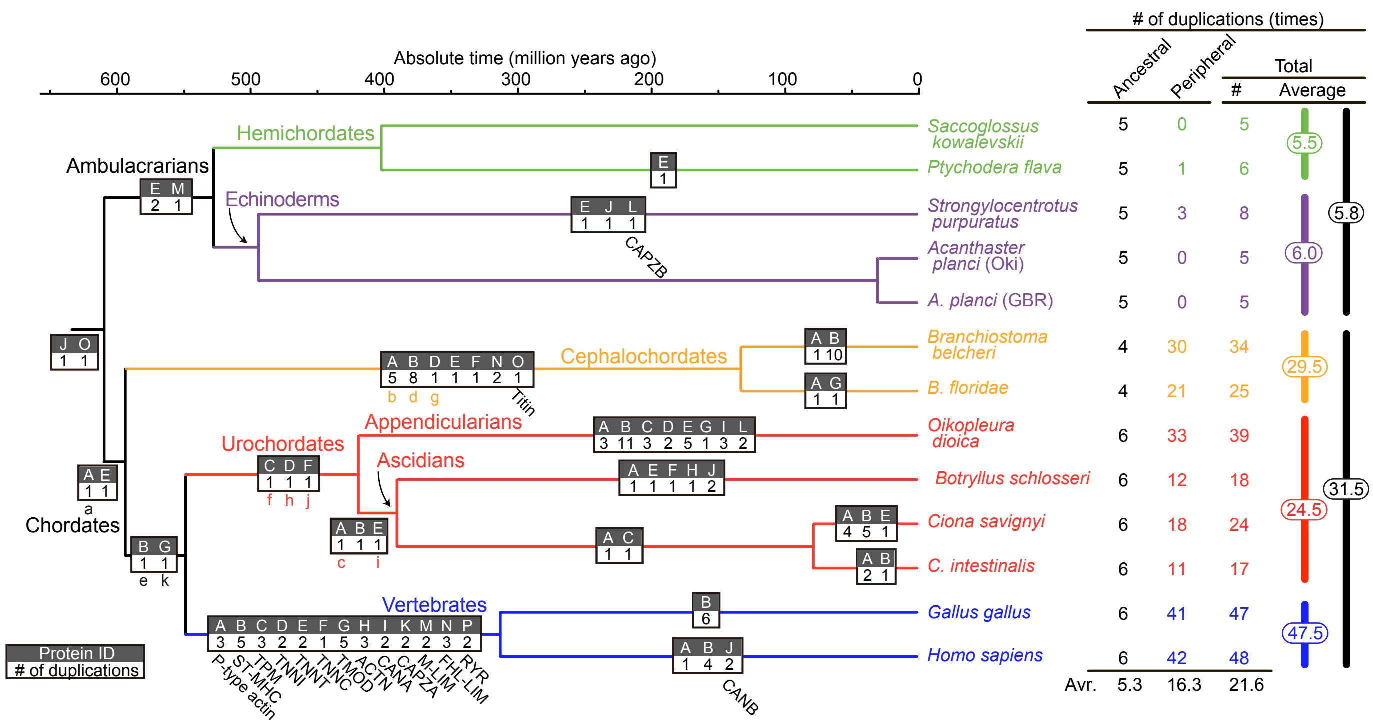

Absolute time (million years ago)
600 500 400 300 200 100 0
# of duplications (times)
Ancestral
Peripheral
Total
#
Average
Ambulacrarians
Hemichordates
Echinoderms
Chordates
Cephalochordates
Urochordates
Appendicularians
Ascidians
Vertebrates
Saccoglossus kowalevskii 5 0 5
Ptychodera flava 5 1 6
Strongylocentrotus purpuratus 5 3 8
Acanthaster planci (Oki) 5 0 5
A. planci (GBR) 5 0 5
Branchiostoma belcheri 4 30 34
B. floridae 4 21 25
Oikopleura dioica 6 33 39
Botryllus schlosseri 6 12 18
Ciona savignyi 6 18 24
C. intestinalis 6 11 17
Gallus gallus 6 41 47
Homo sapiens 6 42 48
Avr. 5.3 16.3 21.6
5.5
6.0
5.8
29.5
24.5
31.5
47.5
Protein ID
# of duplications
J O / 1 1
A E / 1 1 / a
B G / 1 1 / e k
E M / 2 1
E / 1
E J L / 1 1 1
CAPZB
A B D E F N O / 5 8 1 1 1 2 1 / b d g
Titin
A B / 1 10
A G / 1 1
C D F / 1 1 1 / f h j
A B E / 1 1 1 / c i
A B C D E G I L / 3 11 3 2 5 1 3 2
A E F H J / 1 1 1 1 2
A C / 1 1
A B E / 4 5 1
A B / 2 1
A B C D E F G H I K M N P / 3 5 3 2 2 1 5 3 2 2 2 3 2
P-type actin
ST-MHC
TPM
TNNI
TNNT
TNNC
TMOD
ACTN
CANA
CAPZA
M-LIM
FHL-LIM
RYR
B / 6
A B J / 1 4 2
CANB

Fig. S3
